# Transcription Factor NFAT5 Promotes Glioblastoma Cell-driven Angiogenesis via SBF2-AS1/miR-338-3p-Mediated EGFL7 Expression Change

**DOI:** 10.3389/fnmol.2017.00301

**Published:** 2017-09-21

**Authors:** Hai Yu, Jian Zheng, Xiaobai Liu, Yixue Xue, Shuyuan Shen, Lini Zhao, Zhen Li, Yunhui Liu

**Affiliations:** ^1^Department of Neurosurgery, Shengjing Hospital of China Medical University Shenyang, China; ^2^Liaoning Research Center for Clinical Medicine in Nervous System Disease Shenyang, China; ^3^Key laboratory of Neuro-oncology in Liaoning Province Shenyang, China; ^4^Department of Neurobiology, College of Basic Medicine, China Medical University Shenyang, China; ^5^Key Laboratory of Cell Biology, Ministry of Public Health of China, and Key Laboratory of Medical Cell Biology, Ministry of Education of China Shenyang, China

**Keywords:** glioblastoma angiogenesis, transcription factor, long non-coding RNA, NFAT5, SBF2-AS1, miR-338-3p, EGFL7

## Abstract

Glioblastoma (GBM) is the most aggressive primary intracranial tumor of adults and confers a poor prognosis due to high vascularization. Hence anti-angiogenic therapy has become a promising strategy for GBM treatment. In this study, the transcription factor nuclear factor of activated T-cells 5 (NFAT5) was significantly elevated in glioma samples and GBM cell lines, and positively correlated with glioma WHO grades. Knockdown of *NFAT5* inhibited GBM cell-driven angiogenesis. Furthermore, long non-coding RNA SBF2 antisense RNA 1 (SBF2-AS1) was upregulated in glioma samples and knockdown of SBF2-AS1 impaired GBM-induced angiogenesis. Downregulation of NFAT5 decreased SBF2-AS1 expression at transcriptional level. In addition, knockdown of *SBF2-AS1* repressed GBM cell-driven angiogenesis via enhancing the inhibitory effect of miR-338-3p on EGF like domain multiple 7 (EGFL7). *In vivo* study demonstrated that the combination of *NFAT5* knockdown and *SBF2-AS1* knockdown produced the smallest xenograft volume and the lowest microvessel density. NFAT5/SBF2-AS1/miR-338-3p/EGFL7 pathway may provide novel targets for glioma anti-angiogenic treatment.

## Introduction

Glioblastoma (GBM) harbors the highest malignancy of glioma. Despite improvements in therapeutic management, the overall prognosis has remained dismal, with a median survival ranging from 12 to 15 months (Wen and Kesari, 2008). GBM is characterized by its profound vascularization. Deregulated pro-angiogenic factors were produced by GBM cells and secreted into the tumor microenvironment, which stimulates tumor endothelial cell-dependent angiogenesis. Neovascularity brings essential oxygen and nutrition as well as promotes GBM malignancy (Jain et al., 2007). Previous study manifested that microvessel density predicts poor prognosis of glioma patients (Leon et al., 1996) which draws growing attention to anti-angiogenic therapy as an effective therapeutical target of glioma.

Transcription factor nuclear factor of activated T-cells 5 (NFAT5) was originally identified for its involvement in hypertonic kidney inner medulla adaptation (Woo et al., 2002; Lopez-Rodriguez et al., 2004). Accumulated findings have indicated that the expression levels of NFAT5 were aberrant in tumors (Küper et al., 2014; Qin et al., 2017). Moreover, growing publications demonstrated its potential roles in tumor angiogenesis and pro-angiogenic factors regulation (Li et al., 2014; Amara et al., 2016). But the expression levels of NFAT5 in gliomas and its potential role in GBM angiogenesis still remain unclear.

It has been increasingly demonstrated that long non-coding RNAs (lncRNAs) are aberrantly expressed in cancers and involved in cancer progression (Beckedorff et al., 2013). lncRNA SBF2 antisense RNA 1 (SBF2-AS1) was initially identified in non-small cell lung cancer and acts as an oncogene (Lv et al., 2016; Zhao et al., 2016). However, little is known about its expression level and its potential role in GBM. To date, it has been found that lncRNAs may exert their biological function by sponging miRNAs (Tay et al., 2014; Katsushima et al., 2016). miR-338-3p was characterized as a tumor suppressor in GBM (Howe et al., 2017). Meanwhile, ectopic expression of miR-338-3p inhibited angiogenesis in hepatocellular carcinoma (Zhang et al., 2016). *In silico* analysis (starBase v2.0: starbase.sysu.edu.cn), SBF2-AS1 has a putative binding site of miR-338-3p. It is unclear whether SBF2-AS1 interacts with miR-338-3p and affects GBM angiogenesis.

EGF-like domain 7 (EGFL7) is an endothelial cell-derived secreted factor and is associated with vascular tube formation (Parker et al., 2004; Campagnolo et al., 2005). Recent evidence showed that EGFL7 is highly expressed in tumors and promotes tumor angiogenesis (Zhang et al., 2013; Wang F. Y. et al., 2017). *In silico* analysis (target 7.1: http://www.targetscan.org), EGFL7 3′-UTR has putative binding sites of miR-338-3p, which indicates that miR-338-3p may quench EGFL7 activity.

In this study, the expression levels of NFAT5 and SBF2-AS1 were investigated in glioma samples and GBM cell lines. In addition, the roles of NFAT5 and SBF2-AS1 in GBM cell-driven angiogenesis were further explored. Furthermore, NFAT5/SBF2-AS1/miR-338-3p/EGFL7 crosstalk in GBM angiogenesis was revealed. Findings in this study may serve as a potential target for glioma treatment.

## Materials and methods

### Clinical sample

A total of 47 cases paraffin-embedded glioma and five cases normal brain tissues (NBTs) were used for the NFAT5 immunohistochemistry staining. A total of 19 liquid nitrogen-stored glioma samples and 5 NBTs were used for NFAT5 Western blot analysis and SBF2-AS1 quantitative real-time PCR analysis. All specimens were obtained from the Department of Neurosurgery, Shengjing Hospital of China Medical University. NBTs were the rejected material from surgeries of brain trauma and epilepsy. Glioma specimens had confirmed pathological diagnosis and were classified according to the World Health Organization (WHO) criteria by two experienced clinical pathologists in a blinded manner. For the use of the above clinical materials for research purposes, approval from the Hospital Ethical Committee was obtained.

### Immunohistochemistry

All paraffin-embedded specimens were sliced into serial 4 μm sections and sections were labeled with primary antibodies against human NFAT5 (1:100; ab3446, Abcam, Cambridge, UK), followed by incubation with biotinylated secondary antibody included in an immunohistochemical labeling kit (KIT-7780; MaxVision, Fu Zhou, China). The NFAT5 expression was scored according to the proportion of positive cells and the staining intensity by two independent investigators who were blinded to tumor grade. The proportion of positively stained tumor cells was graded for 0 (<10% positive tumor cells), 1(10–50% positive tumor cells), 2 (50–90% positive tumor cells), and 3 (>90% positive tumor cells). The intensity of staining were scored 0 for no staining, 1 for weak staining, 2 for moderate staining, and 3 for strong staining. A combined staining index was calculated by multiplying the proportion of positive staining and the intensity of staining. The stained sections were defined as high expression (staining index>4) or low expression (staining index≤4).

### Cell culture and preparation for glioblastoma (GBM) cell-conditioned medium (GCM)

Human GBM cell lines U87, U118, and human embryonic kidney 293T (HEK293T) cells were purchased from the Shanghai Institutes for Biological Sciences Cell Resource Center (Shanghai, China). Normal human astrocytes (NHA) were obtained from Sciencell Research Laboratories (Carlsbad, CA, USA) and cultured in astrocyte medium (Carlsbad, CA, USA). The human brain microvessel endothelial cell (ECs) line was gifted Dr Couraud (Institute Cochin, Paris, France). U87, U118, and HEK293T cells were cultured in Dulbecco's modified Eagle medium of high glucose supplemented with 10% fetal bovine serum. ECs were cultured as described previously (Guo et al., 2014). All cells were maintained in a humidified incubator at 37°C with 5% CO_2_. For the exposure of cells to hypoxia, U87 and U118 cells were incubated in a hypoxic chamber containing 0.3% O_2_, 5% CO_2_, and 94.7% N_2_. GBM cell-conditioned medium was prepared as described previously (Cai et al., 2017).

### RNA extraction and quantitative reverse transcription-PCR (qRT-PCR)

Total RNA was isolated with Trizol (Life Technologies Corporation). TaqMan PCR (TaqMan MicroRNA Reverse Transcription kit and Taqman Universal Master Mix II, Applied Biosystems) and SYBR Green quantitative PCR (One-Step SYBR PrimeScript RT-PCR Kit, Takara, Dalian, China) were carried out in at least triplicate for the target genes. Expression levels of target genes were determined using the 2^−ΔΔCt^ method and normalized to GAPDH. Expression levels of miR-338-3p were normalized to U6. Probes for TaqMan PCR assays and primer sets for SYBR Green assays are listed in Table S1.

### Cell transfection

The short-hairpin RNA direct against human *NFAT5* (NM_138714.3) gene or *SBF2-AS1* (NR_036485.1) gene was reconstructed in pGPU6/Neo vector (NFAT5 (−)) or pGPU6/Hygro vector (SBF2-AS1(−)) (GenePharma, Shanghai, China), respectively. The empty vectors were used as NCs (NFAT5(−)NC, SBF2-AS1(-)NC). Human *EGFL7* (NM_016215.4) gene coding sequence with or without 3′-UTR was ligated into pIRES2 vector (EGFL7(+)-CDS-3′-UTR, EGFL7(+)-CDS-3′-UTR) (GeneScript, Piscataway, NJ, USA), with the empty vector serving as the NC (EGFL7(+)NC). Stable cell lines were established via Geneticin (G418; Sigma-Aldrich, St Louis, MO, USA) and Hygromycin (Solarbio, China) selection. For transient transfection assays, agomir-338-3p (miR-338-3p(+)), antagomir-338-3p (miR-338-3p (−)), and their NC sequence (miR-338-3p (+)NC and miR-338-3p (−)NC) were synthesized (GenePharma). Cells were collected 48 h after transfection. Sequences of shNFAT5, shSBF2-AS1, and shNC were shown in Table S2. The transfection efficiency of NFAT5, SBF2-AS1, EGFL7, and miR-338-3p was shown in Figure S1.

### Western blot analysis

The cell lysates were extracted from U87, U118, and ECs. Equal amounts of each protein was run on 8% SDS/PAGE gels, transferred to polyvinylidene fluoride membranes and incubated with the following antibodies as primary antibodies: NFAT5 (1:1,000, ab3446; Abcam), EGFL7 (1:200, sc-373898, Santa Cruz Biotechnology, Santa Cruz, CA, USA), ERK (1:1,000, 4685; Cell Signaling Technology, Boston, MA, USA), phosphor-ERK (1:2,000, 4370; Cell Signaling Technology), GAPDH (1:1,000, Santa Cruz Biotechnology, Santa Cruz, CA, USA, sc-32233). β-actin (1:5,000, Proteintech, China). HRP-linked anti-mouse IgG and HRP-linked anti-rabbit IgG antibodies were used as secondary antibodies. Each immunoblot was done at least thrice and the signals were quantified using FluorChem 2.0 software (Alpha Innotech, San Leondro, CA, USA).

### Cell viability analysis

ECs cell viability was determined by the Cell Counting kit-8 (CCK-8) assay (Beyotime Institute of Biotechnology, Jiangsu, China). Cells (2 × 10^3^) were seeded in 96-well plates in triplicate and incubated in GBM cell-conditioned medium for 24 h. Each well was incubated with 10 μl CCK-8 for 2 h, and the absorbance was measured at 450 nm using a spectrophotometer. All experiments were performed at least three times.

### Cell migration assay

ECs migration ability was assessed using 6.5-mm transwell chambers with a pore size of 8 μm (#3422 Costar, Corning, NY, USA). Cells (2 × 10^5^) were suspended in serum-free medium and were seeded into the upper chamber. The lower chamber was filled with 600 μl GBM cell-conditioned medium. Migrated cells were fixed, stained and counted under a microscope. Five randomly chosen fields were counted for each well, and the average cell number was determined. The experiments were performed at least in triplicate.

### Tube formation assay

Prechilled 96-well plates were coated with 100 μl Matrigel (BD Biosciences, Bedford, MA, USA). The plates were then incubated at 37°C for 30 min to allow the matrix to solidify. ECs were suspended in GBM cell-conditioned medium and 4 × 10^4^ cells were added to the plates, followed by incubation at 37°C for 6 h. Tube counts were quantified by Chemi Imager 5500 V2.03 software (Alpha Innotech, San Leondro, CA, USA).

### Enzyme-linked immunosorbent assay (ELISA)

EGFL7 levels in glioma-conditioned medium were measured with ELISA. Briefly, 96-well microplates were coated with 100 μl capture antibody (2 μg/ml) against EGFL7 (Santa Cruz Biotechnology), incubated overnight at 4°C. The wells were then blocked with 5% BSA in PBS for 1 h at room temperature. Then samples were applied to wells and incubated for 1 h at room temperature. After washed with PBS with 0.1% Tween-20, horseradish peroxidase conjugated goat anti-mouse immunoglobulin G was added to each well and incubated for 1 h at room temperature. Subsequently, tetramethylbenzidine (TMB) solution was added to the wells, then reaction was stopped by applying sulfuric acid. The absorbance was then measured at 450 nm on a spectrophotometer.

### RNA immunoprecipitation (RIP)

RIP was performed using a Magna RNA-binding protein immunoprecipitation kit (Millipore, Billerica, MA, USA) following the manufacturer's instructions. U87 and U118 cell lysates containing SBF2-AS1 and miR-338-3p were prepared and incubated with anti-argonaute2 (Ago2) antibody (Millipore). Normal mouse IgG (Millipore) was used as a NC. SBF2-AS1 and miR-338-3p present in the precipitates were assayed by qRT-PCR.

### Luciferase reporter assay

HEK293T cells were seeded into a 96-well plate. A wild-type and mutated SBF2-AS1 (SBF2-AS1-Wt and SBF2-AS1-Mut containing an 8 bp mutation in the predicted binding sites of miR-338-3p) or EGFL7 3′-UTR (EGFL7-Wt, EGFL7-Mut1, EGFL7-Mut2, and EGFL7-Mut3 containing individual or combined mutation in the predicted binding sites of miR-338-3p) luciferase reporter gene vector were constructed. The pmirGLO dual-luciferase vector (Promega, Madison, WI, USA) was used as a control. Cells were co-transfected with the indicated vectors and agomir-338-3p and agomir-338-3p-NC, respectively. Luciferase assays were performed 48 h after transfection using the Dual Luciferase Reporter Assay System (Promega).

### Chromatin immunoprecipitation (ChIP) assay

ChIP assay was performed with the Simple Chip Enzymatic Chromatin IP kit (Cell Signaling Technology, Danvers, MA, USA) as described previously (Yu et al., 2017). Briefly, U87 and U118 chromatin were incubated with 3 μg anti-NFAT5 (ab3446, Abcam). Purified immunoprecipitated DNA was prepared for the PCR with the specific primers. The primers used for PCR are presented in Table S3.

### Nude mouse xenograft model

All animal experiments were complied with the guidelines of the Animal Welfare Act and were reviewed and approved by the Ethics Committee of Shengjing Hospital. Four weeks old female BALB/C nude mice were purchased from Cancer Institute of the Chinese Academy of Medical Science. Mice were randomly divided into each double-blind group by two performers, *n* = 7 per group. A suspension of 5 × 10^5^ cells in a 100 μl volume was injected subcutaneously into the right flank of the mice. Tumor nodules were estimated with caliper at a 5-day interval. Mice were scarified on the 45th day after injection, and the tumors were excised and collected for the CD31 staining. Tumor volumes = (length × width^2^)/2.

### Microvessel density (MVD) assay

MVD of xenograft gliomas were determined by the immunohistochemical staining of endothelial marker CD31 and evaluated according to the previous description (Weidner et al., 1991; Cai et al., 2017). Areas of highest vascularization were selected by scanning the tumor sections at low magnification. Stained microvessels were counted in a single 200 × field within the selected filed by three observers without previous knowledge of tumor groups. The following cellular structures were considered as countable microvessel: (a) stained lumen, (b) stained endothelial cell, (c) stained endothelial cell cluster (a and b are clearly separated from adjacent strained lumens, tumor cells and other connective tissue elements). The MVD value was calculated as the average vessel counts in three selected areas within a microscopic field.

### Statistical analysis

All statistical analyses were performed with GraphPad Prism 5 (GraphPad Software, La Jolla, CA, USA). All values are presented as the mean± standard deviation (*SD*) from at least three independent experiments. Student's *t*-test was used for comparisons between two groups. One-way ANOVA was used for multi-group comparisons followed by Bonferroni *post-hoc* test. A chi-square test was applied to determine the association of NFAT5 levels with WHO grades. The difference was considered statistically significant when *P* < 0.05.

## Results

### NFAT5 expression positively correlated with glioma grade

**Figure 1:**
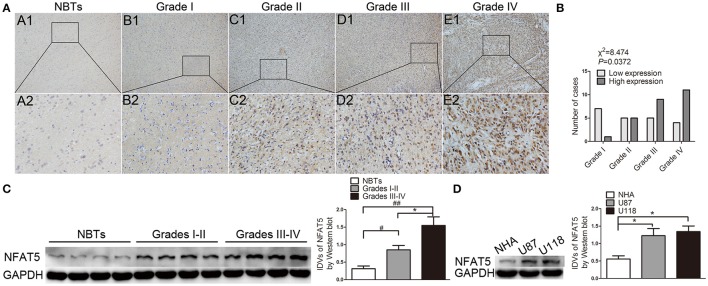
NFAT5 was upregulated in glioma tissues and GBM cell lines. **(A)** Representative patterns of NFAT5 expression levels that were determined by immunohistochemistry staining in glioma tissues and normal brain tissues. A1–A2 normal brain tissues (*n* = 5), B1–B2 grade I gliomas (*n* = 8), C1–C2 grade II gliomas (*n* = 10), D1–D2 grade III gliomas (*n* = 14), E1–E2 grade IV gliomas (*n* = 15). Original magnification, × 100 in A1–E1, × 400 in A2–E2. **(B)** Association of NFAT5 expression levels with WHO grade. *P* value was estimated by chi-square test. **(C)** Representative patterns of NFAT5 expression levels that were detected by Western blot in glioma tissues (Grades I–II, 7 cases; Grades III–IV, 12 cases) and NBTs (5 cases). Data represent mean ± s.d. (each case was determined by Western blot for three times), ^#^*P* and ^*^P < 0.05, ^##^*P* < 0.01. **(D)** Expression of NFAT5 was detected by Western blot in normal human astrocytes, U87 and U118 cell lines. Data represent mean ± s.d. (*n* = 3, each). ^*^P < 0.05.

Immunohistochemistry results showed that NBTs presented weak NFAT5 staining. However, the proportion of NFAT5-positive cells and the staining intensity were higher in glioma sections (Figure 1A) and presented a tumor pathological grade-dependent pattern (Figure 1B). Consistent with the immunohistochemistry results, Western blot assay showed that NFAT5 levels were elevated in glioma tissues and were correlated with the pathological grade (Figure 1C). In addition, compared with NHA, significant upregulation of NFAT5 was observed in U87 and U118 glioblastoma (GBM) cell lines (Figure 1D). It is well-established that hypoxia plays an important role for GBM angiogenesis. Therefore, we tested the NFAT5 levels under the hypoxic conditions. As shown in Figure S1 NFAT5 levels were upregulated in U87 and U118 cells under hypoxic conditions compared with under normoxic conditions.

### Knockdown of *NFAT5* suppressed GBM cell-driven angiogenesis *in vitro*

**Figure 2:**
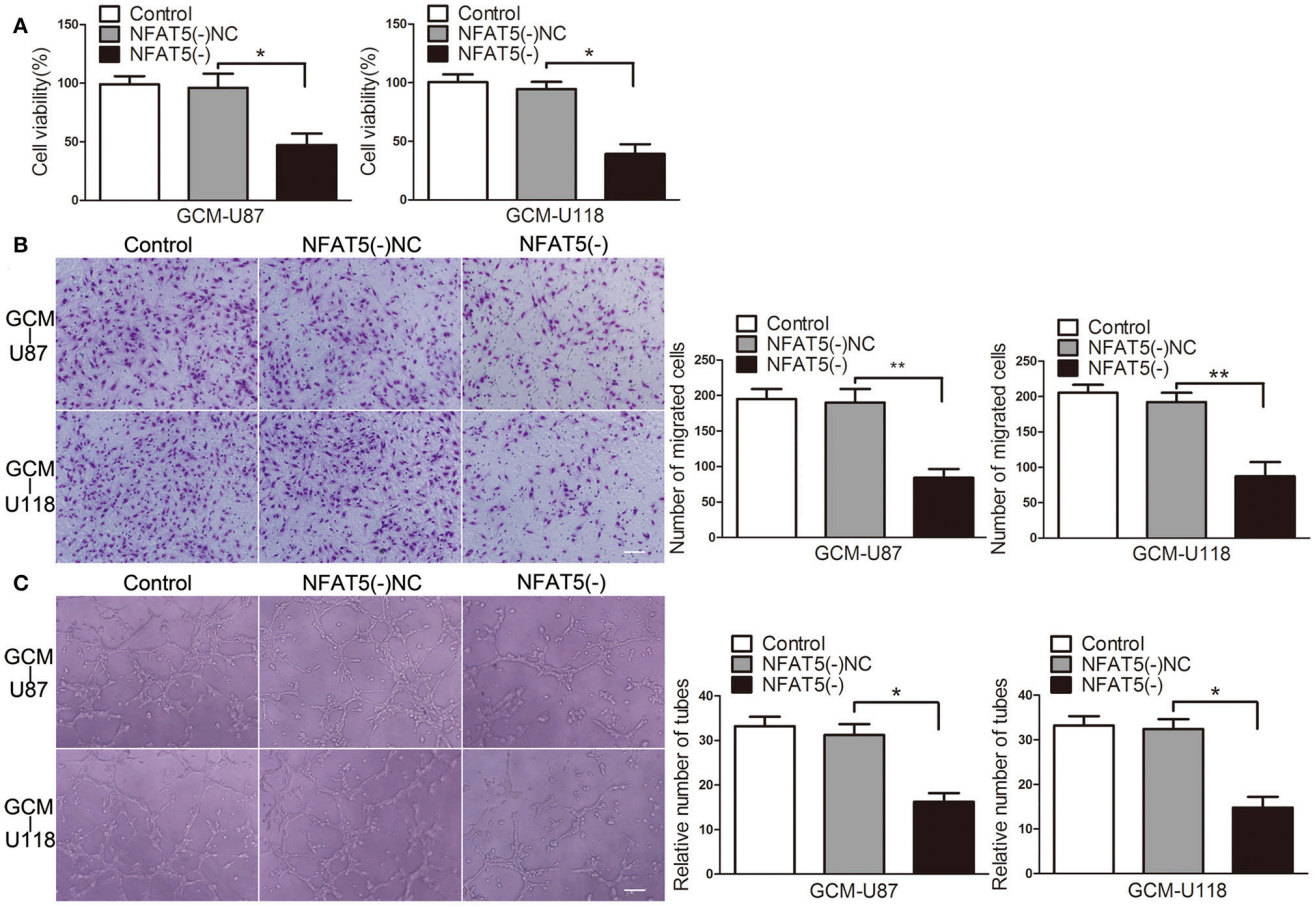
Knockdown of *NFAT5* suppressed GBM cell-driven angiogenesis *in vitro. NFAT5* was knockdown in U87 and U118 cells. Effects of GBM cell-conditioned medium on ECs were detected. **(A)** ECs cell viability was measured by CCK-8 assay. **(B)** ECs migration was measured by Transwell assay. **(C)** ECs tube formation was measured by Matrigel tube formation assay. Data represent mean ± s.d. (*n* = 4, each). ^*^P < 0.05, ^**^P < 0.01. Scale bar represents 30 μm.

Since GBM cells have a strong capacity for stimulating angiogenesis, we used GBM cell-conditioned medium as a means to induce angiogenesis in human brain microvessel endothelial cells to determine *in vitro* angiogenesis ability as previous description (Wurdinger et al., 2008). In order to characterize the influence of NFAT5 on GBM cell-driven angiogenesis, stable *NFAT5* knockdown U87 and U118 cells were conducted. Then, effects of GBM cell-conditioned medium on endothelial cells (ECs) were tested. As shown in Figure 2A, there was no significant difference in ECs cell viability between the control group and the *NFAT5* knockdown negative control (NFAT5(-)NC) group. However, cell viability was significantly decreased in the *NFAT5* knockdown (NFAT5(-)) group compared with that in the NFAT5(-)NC group. Similarly, knockdown of *NFAT5* inhibited the migration (Figure 2B) and tube formation (Figure 2C) of ECs. The above data indicated that downregulation of NFAT5 suppressed GBM cell-driven angiogenesis *in vitro*.

### SBF2-AS1 was upregulated in gliomas and knockdown of SBF2-AS1 inhibited GBM cell-driven angiogenesis *in vitro*

**Figure 3:**
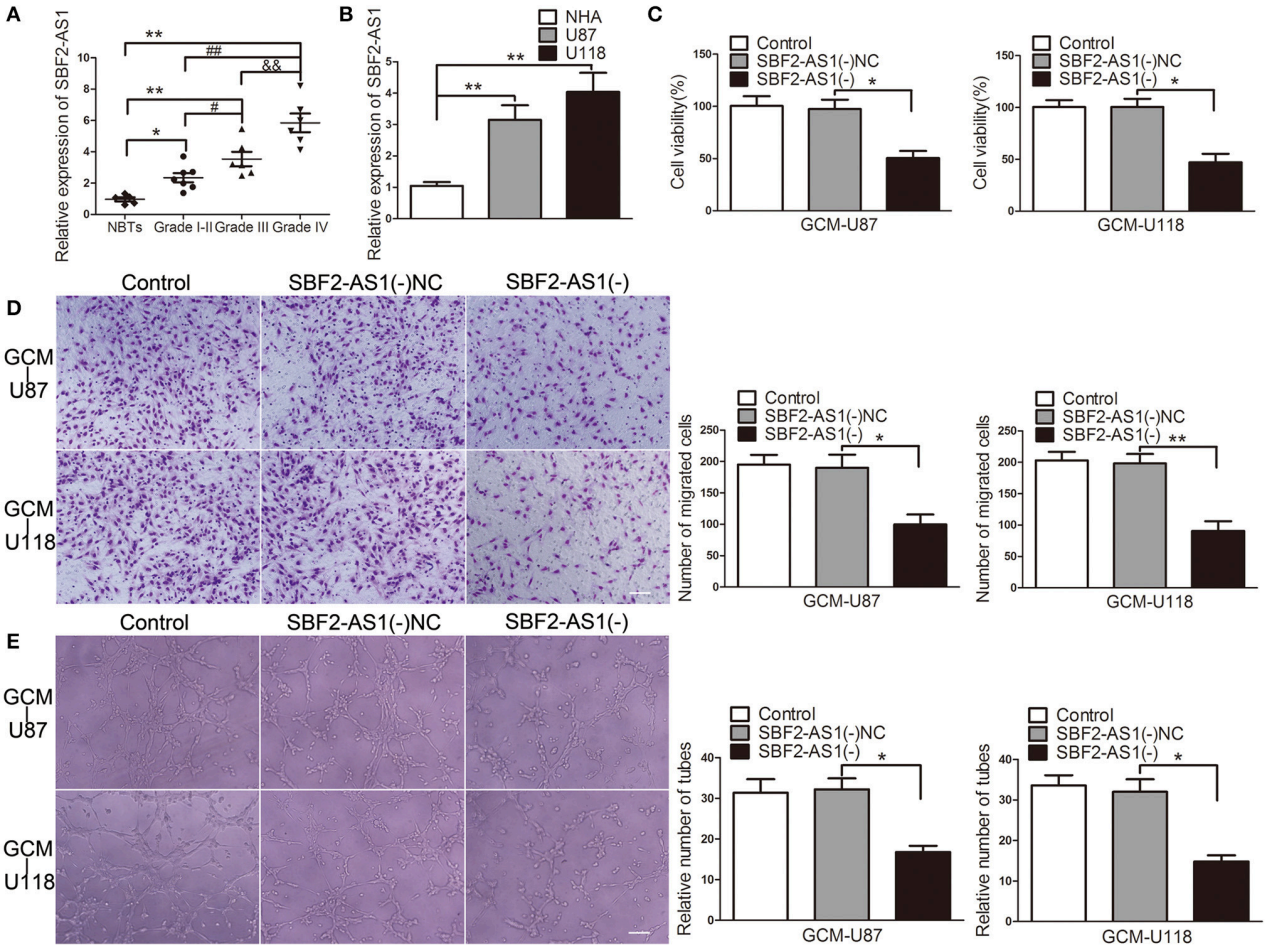
Downregulation of SBF2-AS1 inhibited GBM cell-driven angiogenesis *in vitro*. **(A)** Expression levels of SBF2-AS1 were detected by qRT-PCR in glioma tissues (Grades I–II, seven cases; Grade III, six cases; Grade IV, six cases) and NBTs (five cases). Data represent mean ± s.d. (*n* = 3, each), ^#^*P* and ^*^P < 0.05,^**^P, ^&&^*P*, and ^##^*P* < 0.01. **(B)** Expression levels of SBF2-AS1 were detected by qRT-PCR in normal human astrocytes, U87 and U118 cell lines. Data represent mean ± s.d. (*n* = 4), ^**^P < 0.01. *SBF2-AS1* was knockdown in U87 and U118 cells. Effects of GBM cell-conditioned medium on ECs were detected. **(C)** ECs cell viability was measured by CCK-8 assay. **(D)** ECs migration was measured by Transwell assay. **(E)** ECs tube formation was measured by Matrigel tube formation assay. Data represent mean ± s.d. (*n* = 4, each). ^*^P < 0.05, ^**^P < 0.01. Scale bar represents 30 μm.

Long non-coding RNAs (lncRNAs) are important mediators for tumor angiogenesis. SBF2-AS1 is an lncRNA that was proved to be upregulated in non-small cell lung cancer (Lv et al., 2016). Expression levels of SBF2-AS1 in gliomas and its potential roles in GBM cell-driven angiogenesis were unclear. As shown in Figure 3A, SBF2-AS1 was upregulated in glioma tissues and was positively correlated with pathological grade. In addition, compared with in NHA, SBF2-AS1 levels were increased in U87 and U118 cells (Figure 3B). To clarify the potential role of SBF2-AS1 in GBM cell-driven angiogenesis, we next examined the effects of SBF2-AS1 silencing on GBM cell-driven angiogenesis. As shown in Figures 3C–E, ECs were suppressed with supernatant from *SBF2-AS1* knockdown GBM cells leading to a significant reduction of cell viability, migration, and tube formation. These results revealed that SBF2-AS1 inhibition impaired GBM cell-driven angiogenesis.

### NFAT5 regulated *SBF2-AS1* at transcriptional level, and SBF2-AS1 was involved in NFAT5-mediated GBM cell-driven angiogenesis

**Figure 4:**
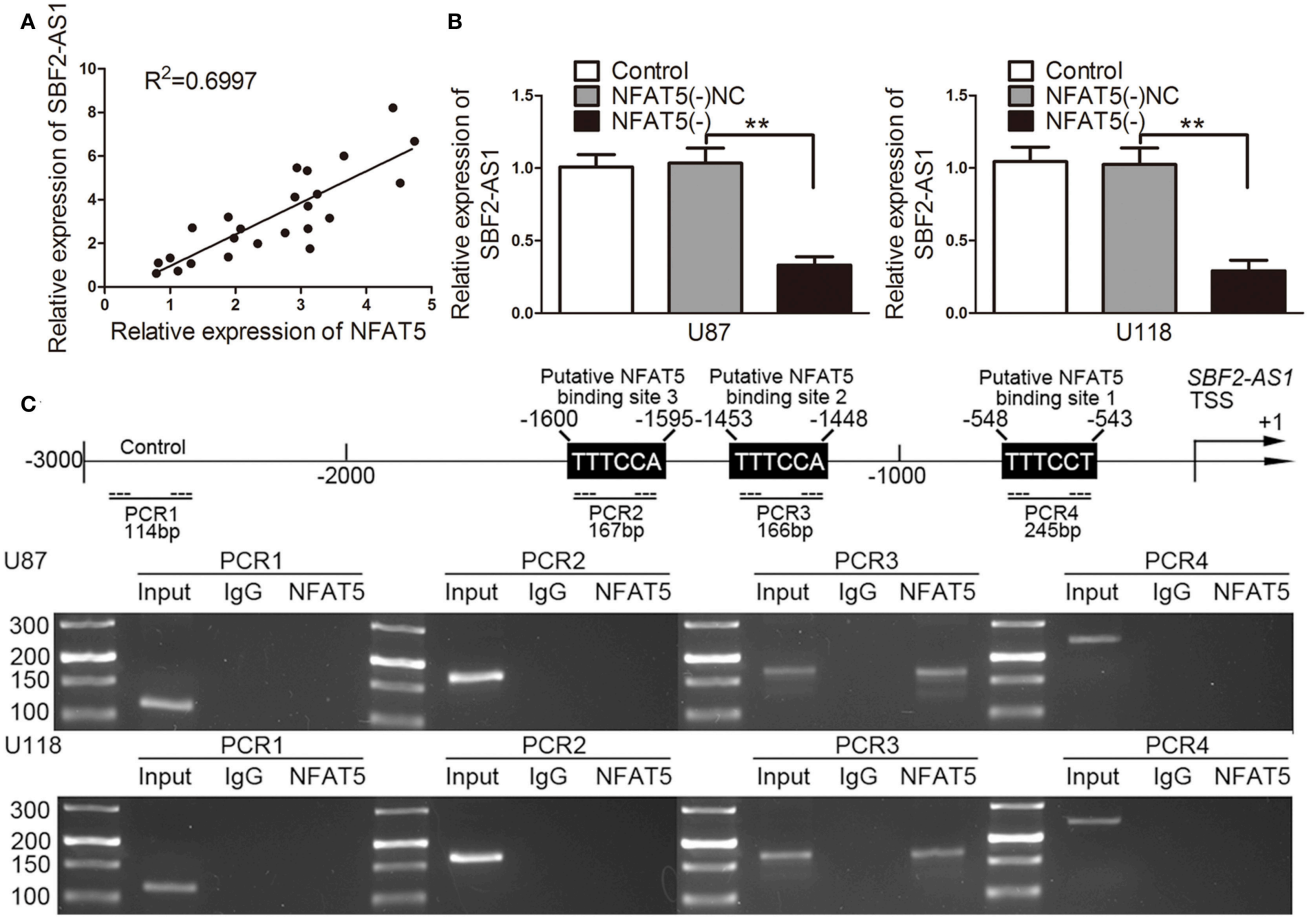
NFAT5 regulated SBF2-AS1 at transcriptional level. **(A)** Linear regression analysis was performed to each individual NFAT5 and SBF2-AS1 expression, *P* < 0.01. **(B)** NFAT5 was knockdown in U87 and U118 cells. SBF2-AS1 levels in U87 and U118 cells were measured by qRT-PCR. Data represent mean ± s.d. (*n* = 4, each). ^**^P < 0.01. **(C)** Schematic representation of the human *SBF2-AS1* promoter region. Chromatin immunoprecipitation PCR products for putative NFAT5-binding sites and an upstream region not expected to associate with NFAT5 are amplified by PCR using their specific primers (*n* = 3, each).

The correlation analysis of NFAT5 and SBF2-AS1 showed that SBF2-AS1 level was positively correlated with NFAT5 level (Figure 4A). Thus, we hypothesized that SBF2-AS1 might be involved in NFAT5-mediated GBM cell-driven angiogenesis. To confirm the hypothesis, we detected SBF2-AS1 levels in *NFAT5* knockdown U87 and U118 cells by qRT-PCR. As shown in Figure 4B, knockdown of *NFAT5* significantly decreased SBF2-AS1 levels in U87 and U118 cells. Transcription factors may bind to the specific region of promoters to regulated gene expression. TGGAAA is the consensus binding sequence of NFAT5 (Kultz and Csonka, 1999). Combined with scanning the *SBF2-AS1* promoter region by using the JASPAR database (http://jaspar.binf.ku.dk/), we found three putative NFAT5 binding sites (Figure 4C). Then chromatin immunoprecipitation assay was performed to clarify the association of NFAT5 and *SBF2-S1* promoter region. As shown in Figure 4C primers were designed to amplify the 2,000 bp upstream region of the putative NFAT5 binding site that was not expected to associate with NFAT5. PCR products were observed in the NFAT5 immunoprecipitation in the PCR3 group, which indicated that NFAT5 bound to *SBF2-AS1* promoter at –1,453 to –1,448 bp region. The above data indicated that NFAT5 regulated *SBF2-AS1* at transcriptional level.

**Figure 5:**
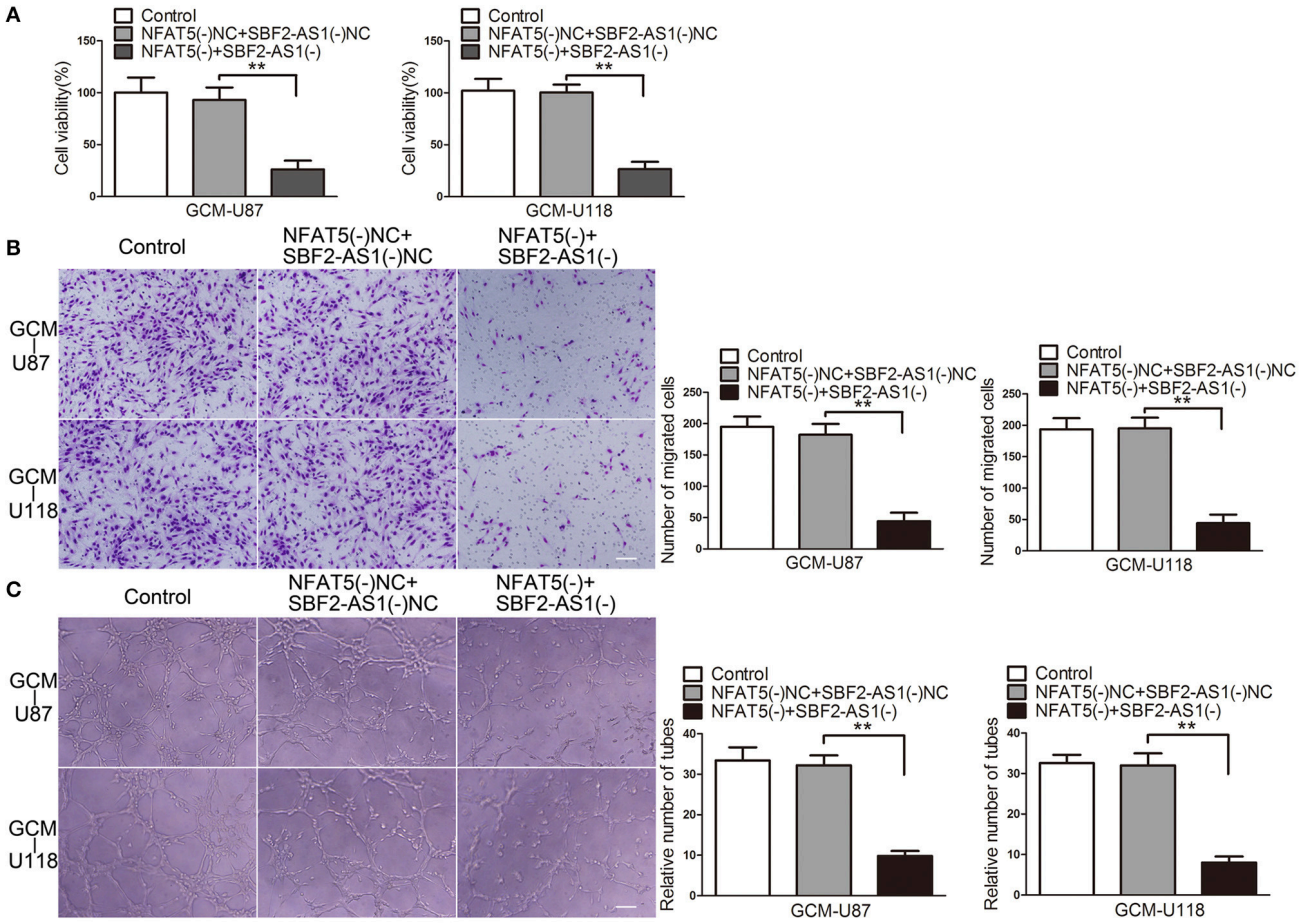
Combination of *NFAT5* and *SBF2-AS1* knockdown inhibited GBM cell-driven angiogenesis. *NFAT5* and *SBF2-AS1* were knockdown in U87 and U118 cells. Effects of NFAT5 and SBF2-AS1 on ECs cell viability **(A)**, migration **(B)**, and tube formation **(C)** were measured. Data represent mean ± s.d. (*n* = 4). ^**^P < 0.01. Scale bar represents 30 μm.

Moreover, the combination of *NFAT5* and *SBF2-AS1* knockdown enhanced the inhibitory effects on ECs cell viability (Figure 5A), migration (Figure 5B), and tube formation (Figure 5C), which were induced by *NFAT5* knockdown alone.

The above findings indicated that SBF2-AS1 might be involved in NFAT5-mediated GBM cell-driven angiogenesis.

### SBF2-AS1 sponged miR-338-3p and miR-338-3p was a mediator of SBF2-AS1-regualted GBM cell-driven angiogenesis

**Figure 6:**
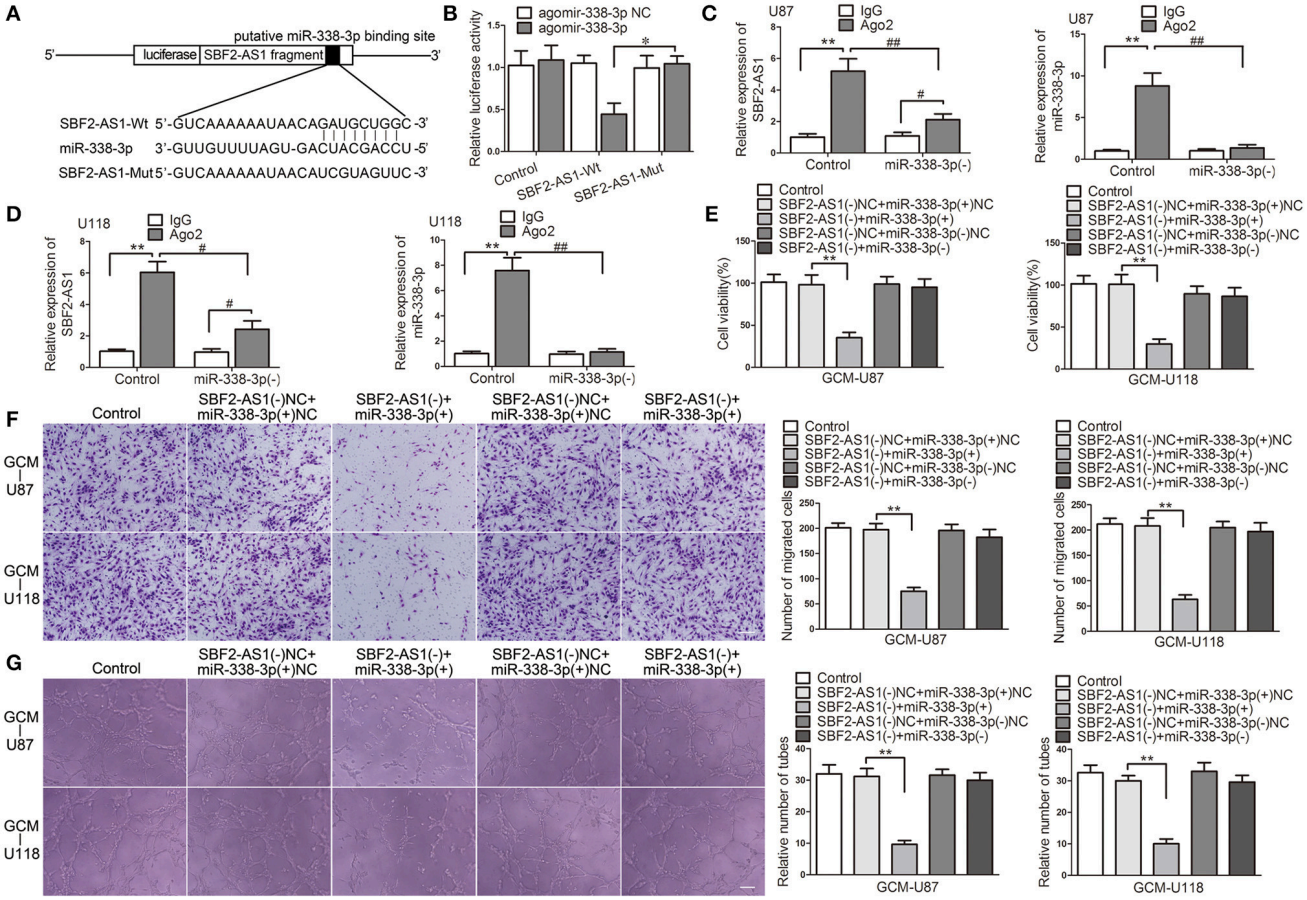
miR-330-3p was sponged by SBF2-AS1 and was involved in SBF2-AS1-mediated GBM cell-driven angiogenesis. **(A)** Luciferase reporter vectors that carry cDNA sequences of *SBF2-AS1* containing the wild-type or mutant miR-338-3p binding site and the seed region of miR-338-3p. **(B)** Relative luciferase activity in HEK293T cells which were co-transfected pmirGLO-SBF2-AS1-Wt or Mut and agomir-338-3p were determined. Data represent mean ± s.d. (*n* = 4, each). ^*^P < 0.05. **(C,D)** RNA immunoprecipitation assay was performed with normal mouse IgG or anti-Ago2 in U87 and U118 cells. Relative enrichment of SBF2-AS1 and miR-338-3p were determined by qRT-PCR. Data represent mean ±s.d. (*n* = 4, each). ^#^*P* < 0.05, ^##^*P*, and ^**^P < 0.01. Agomir-338-3p or antagomir-338-3p was transfected in cells that SBF2-AS1 was stably knocked down. Effects of co-transfection of SBF2-AS1 and miR-338-3p on ECs cell viability **(E)**, migration **(F)**, and tube formation **(G)** were measured. Data represent mean ± s.d. (*n* = 4, each), ^**^P < 0.01. Scale bar represents 30 μm.

*In Silico* analysis, SBF2-AS1 has a potential miR-338-3p binding site. Thus, we hypothesized that SBF2-AS1 may regulate GBM cell-driven angiogenesis by sponging miR-338-3p. To confirm the hypothesis, we performed the dual luciferase reporter assay and RNA immunoprecipitation (RIP) assay. As shown in Figure 6A, luciferase reporter plasmids ligating cDNA sequences of *SBF2-AS1* containing the wild-type or mutant miR-338-3p binding site were conducted. Co-transfection of the luciferase reporter conduct containing the wild-type, but not the mutant SBF2-AS1 sequence with agomir-338-3p resulted in decreased luciferase activity compared with agomir-338-3p NC (Figure 6B). RIP assay using antibody against Ago2 showed higher SBF2-AS1 and miR-338-3p levels in the Ago2 precipitates compared with the IgG precipitates. Moreover, knockdown of miR-338-3p decreased the enrichment of SBF2-AS1 and miR-338-3p in Ago2 precipitates (Figures 6C,D). The above data indicated that SBF2-AS1 sponged miR-338-3p in a sequence-dependent manner.

Having found that miR-338-3p was regulated by SBF2-AS1, we speculated that it might participate in SBF2-AS1-mediated GBM cell-driven angiogenesis. To test this, miR-338-3p was overexpressed or knocked down in GBM cells with SBF2-AS1 silencing. Then effects of the GBM cell supernatant on ECs were determined. As shown in Figure 6E, overexpression of miR-338-3p promoted the suppressive effect on EC cell viability which was induced by SBF2-AS1 knockdown. While downregulation of miR-338-3p reversed the effect induced by SBF2-AS1 inhibition. Similar effects were observed on ECs migration (Figure 6F) and tube formation (Figure 6G).

Collectively, these results supported an involvement of miR-338-3p in SBF2-AS1-mediated GBM cell-driven angiogenesis.

### miR-338-3p upregulation suppressed EGFL7-induced pro-angiogenic effect by targeting EGFL7 3′-UTR

**Figure 7:**
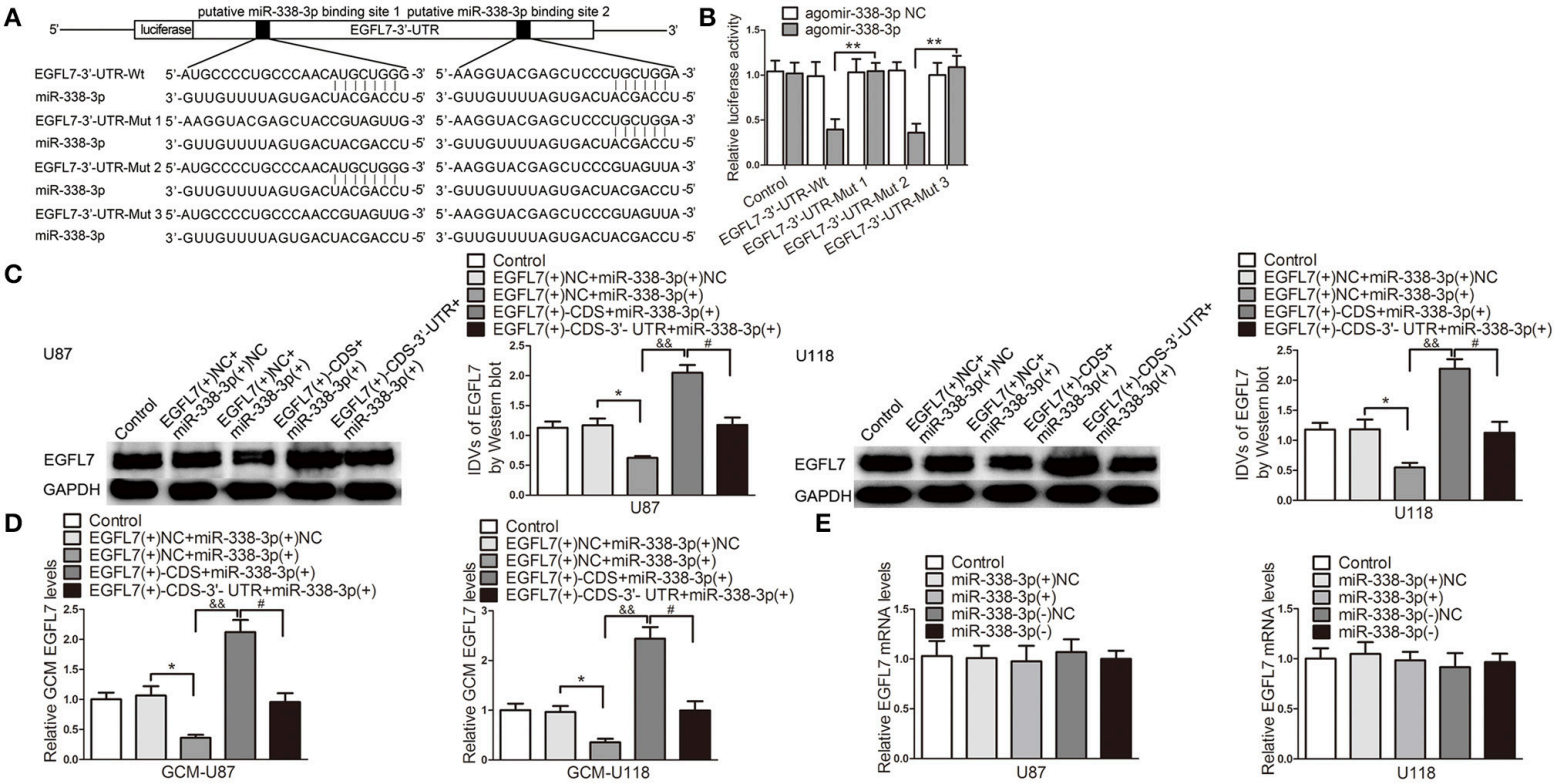
miR-338-3p downregulated EGFL7 by targeting its 3′-UTR. **(A)** The binding site on EGFL7 3′-UTR and the seed region of miR-338-3p. **(B)** Relative luciferase activity in HEK293T cells that co-transfected pmirGLO-EGFL7-3′-UTR-Wt or Muts and agomir-338-3p were determined. Data represent mean ± s.d. (*n* = 3, each). ^**^P < 0.01. Agomir-338-3p was transiently transfected in U87 and U118 cells that were stably overexpressed EGFL7 with or without 3′-UTR. Effects of co-transfection of agomir-338-3p and EGFL7 with or without 3′-UTR on EGFL7 protein level **(C)**, secretion **(D)** were measured. Effect of miR-338-3p on EGFL7 mRNA level **(E)** was measured. Data represent mean ± s.d. (*n* = 4). ^*^P and ^#^*P* < 0.05, ^&&^*P* < 0.01.

To perfect the precise mechanism which may be involved in miR-338-3p-regulated GBM cell-driven angiogenesis, we screened EGFL7 out as a potential mediator. miRNAs target the 3′-UTR of mRNAs to degrade mRNA or inhibit translation. We next performed the dual luciferase reporter assay to clarify the association of miR-338-3p and EGFL7 3′-UTR. Figure 7A is the schematic representation of luciferase reporter vectors carrying wild-type or mutant EGFL7 3′-UTR. In HEK293T cells, miR-338-3p effectively reduced the relative luciferase activity of the EGFL7 3′-UTR-Wt conduct and EGFL7 3′-UTR-Mut2, but not that of the EGFL7 3′-UTR-Mut1 and EGFL7 3′-UTR-Mut3 conducts (Figure 7B), indicating that the putative binding site 1 was functional. To further confirm the above funding, miR-338-3p was overexpressed in GBM cells which were stably transfected with pIRES2/EGFL7-CDS or pIRES2/EGFL7-CDS-3′-UTR. Western blot results showed that EGFL7 levels in U87 and U118 cells were lower in EGFL7(+)-CDS-3′-UTR+miR-338-3p group than that in EGFL7(+)-CDS+miR-338-3p group (Figure 7C). Similarly, EGFL7 levels in U87 and U118 cell conditioned-medium was decreased in EGFL7(+)-CDS-3′-UTR+miR-338-3p group compared with in EGFL7(+)-CDS+miR-338-3p group (Figure 7D). However, upregulation or downregulation of miR-338-3p had little effect on EGFL7 mRNA level (Figure 7E). The above data indicated that miR-338-3p targeted the EGFL7 3′-UTR to decrease *EGFL7* expression via translation inhibition.

**Figure 8:**
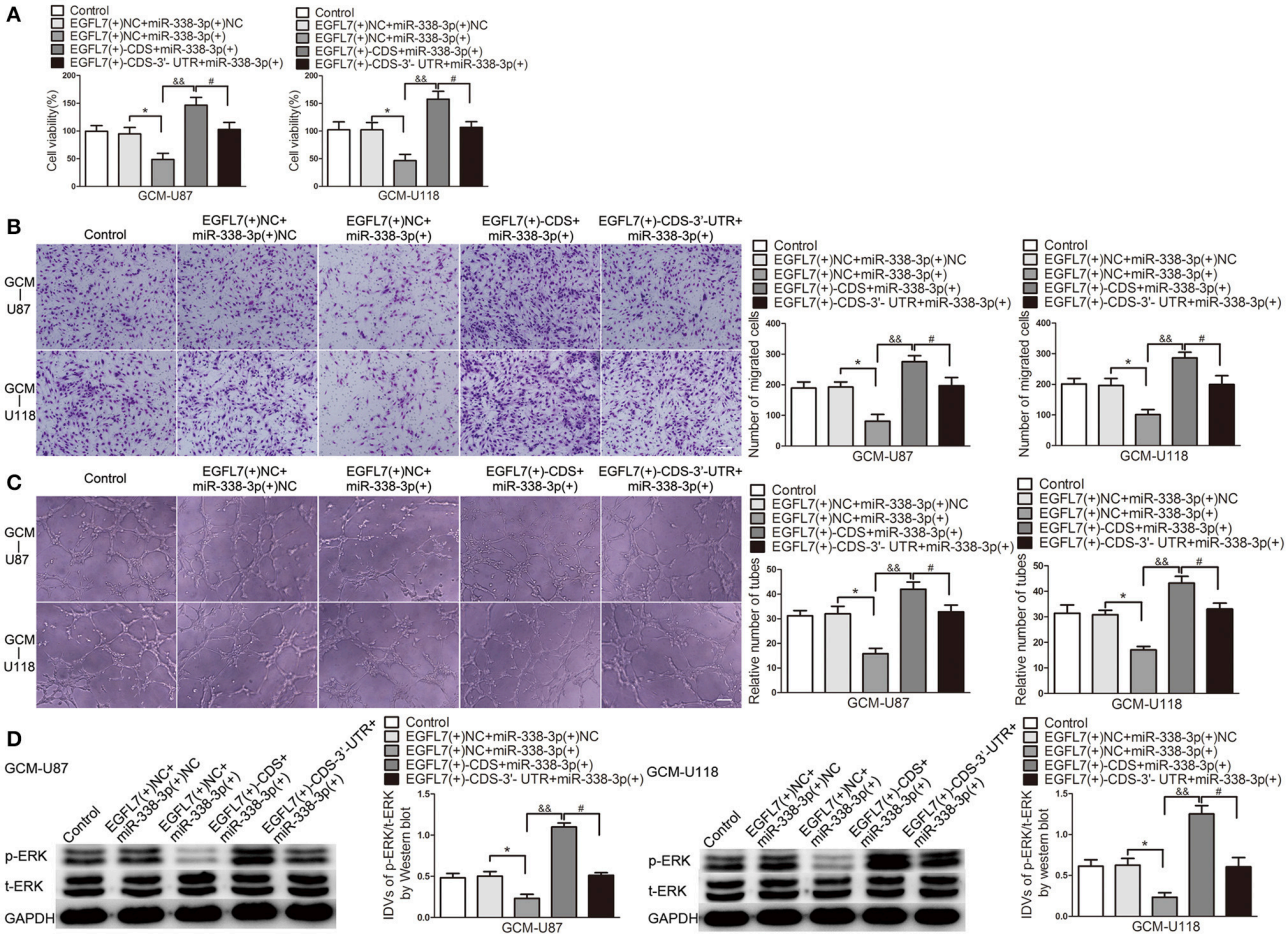
miR-338-3p suppressed EGFL7-induced pro-angiogenic effect by targeting EGFL7 3′-UTR. **(A)** Effects of co-transfection of agomir-338-3p and EGFL7 with or without 3′-UTR on ECs cell viability **(B)**, migration **(C)**, and tube formation **(D)** were measured. Effect of co-transfection of agomir-338-3p and EGFL7 with or without 3′-UTR on ERK levels was measured by Western blot. Data represent mean ± s.d. (*n* = 4). ^*^P and ^#^*P* < 0.05, ^&&^*P* < 0.01. Scale bar represents 30 μm.

Similar to the above Western blot and ELISA results, ECs cell viability (Figure 8A), migration (Figure 8B), and tube formation (Figure 8C) was significantly reduced in EGFL7(+)-CDS-3′-UTR+miR-338-3p group compared with that in EGFL7(+)-CDS+miR-338-3p group.

EGFL7 exerts pro-angiogenic by activating ERK pathway. Therefore, we detected p-ERK level in human brain microvessel ECs treated with related GBM cell-conditioned medium. As shown in Figure 8D, p-ERK/t-ERK was decreased in EGFL7(+)-CDS-3′-UTR+miR-338-3p group compared with that in EGFL7(+)-CDS+miR-338-3p group. The above date indicated that EGFL7 promoted GBM cell-driven angiogenesis at least partially by activation of ERK pathway in ECs.

### Knockdown of *NFAT5* and *SBF2-AS1* inhibited EGFL7 expression and secretion without affecting EGFL7 mRNA

**Figure 9:**
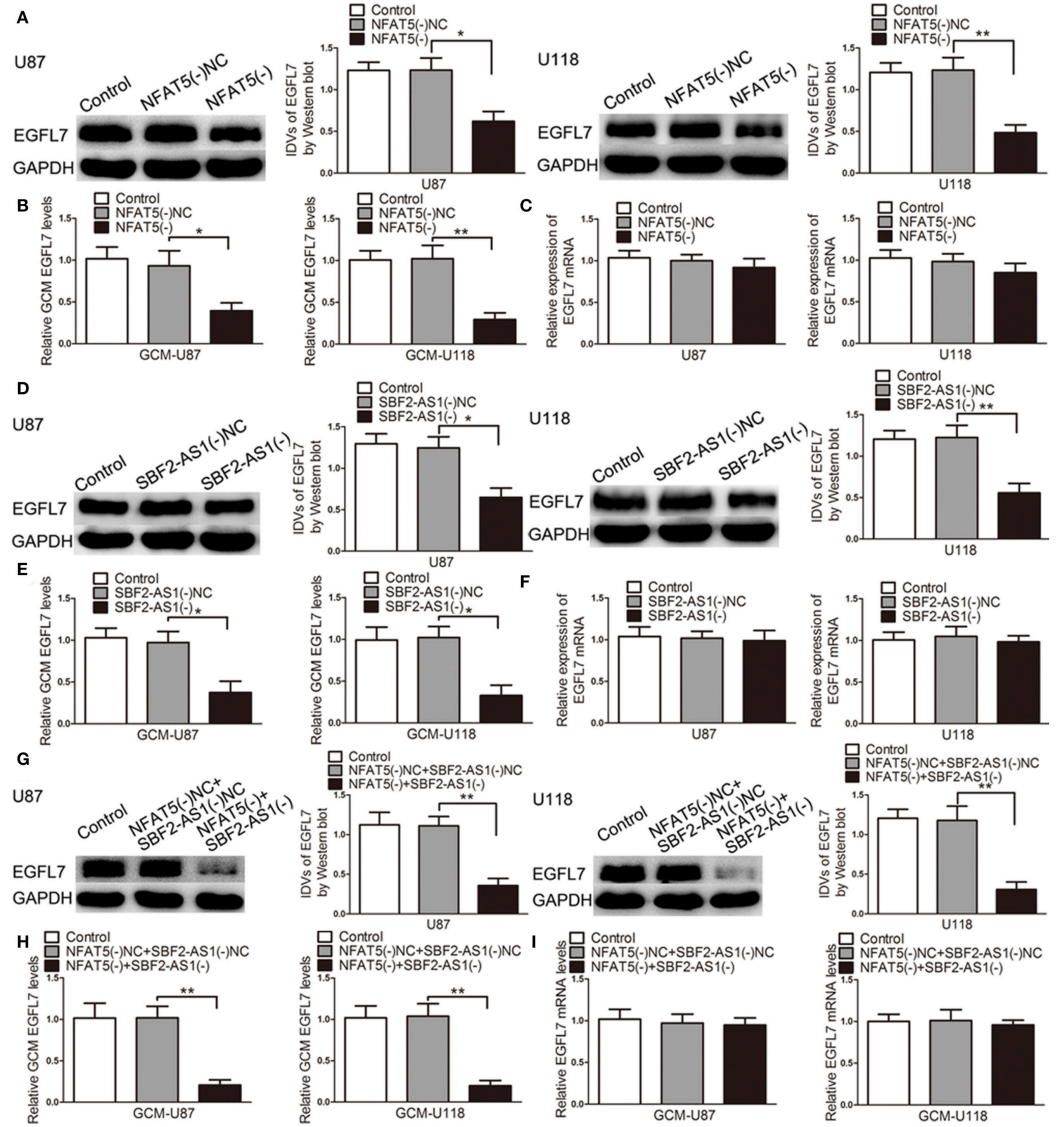
Knockdown of *NFAT5* and *SBF2-AS1* inhibited EGFL7 protein level and secretion without affecting EGFL7 mRNA level. Effects of *NFAT5* knockdown on EGFL7 protein level **(A)**, secretion **(B)**, and mRNA level **(C)**. Effects of *SBF2-AS1* knockdown on EGFL7 protein level **(D)**, secretion **(E)**, and mRNA level **(F)**. Effects of combination knockdown of *NFAT5* and *SBF2-AS1* on EGFL7 protein level **(G)**, secretion **(H)**, and mRNA level **(I)**. Data represent mean ± s.d. (*n* = 4). ^*^P < 0.05, ^**^P < 0.01.

After confirming that EGFL7 was involved in miR-338-3p-regulated GBM cell-driven angiogenesis, we hypothesized that EGFL7 was a mediator of NFAT5/SBF2-AS1-regualted GBM cell-driven angiogenesis. As shown in Figure 9A, EGFL7 expression levels were lower in the NFAT5(-) group than that in the NFAT5(-)NC group. In addition EGFL7 levels were decreased in the NFAT5(-) GBM cell-conditioned medium (Figure 9B). However, there was no significant difference in the EGFL7 mRNA levels between the NFAT5(-)NC group and NFAT5(-) group (Figure 9C). ChIP assay showed that NFAT5 did not associate with the putative binding site in *EGFL7* promoter region (Figure S2), which indicated that NFAT5 might regulate EGFL7 in an indirect way. Similarly, SBF2-AS1 inhibition decreased EGFL7 expression (Figure 9D) and secretion (Figure 9E) without affecting EGFL7 mRNA level (Figure 9F). Moreover, the combination of *NFAT5* and *SBF2-AS1* knockdown enhanced the inhibitory effects induced by individual knockdown (Figures 9G,H). Also, EGFL7 mRNA levels were not changed (Figure 9I).

### *NFAT5* knockdown combined with *SBF2-AS1* knockdown produced the optimum tumor suppressive effect *in vivo*

**Figure 10:**
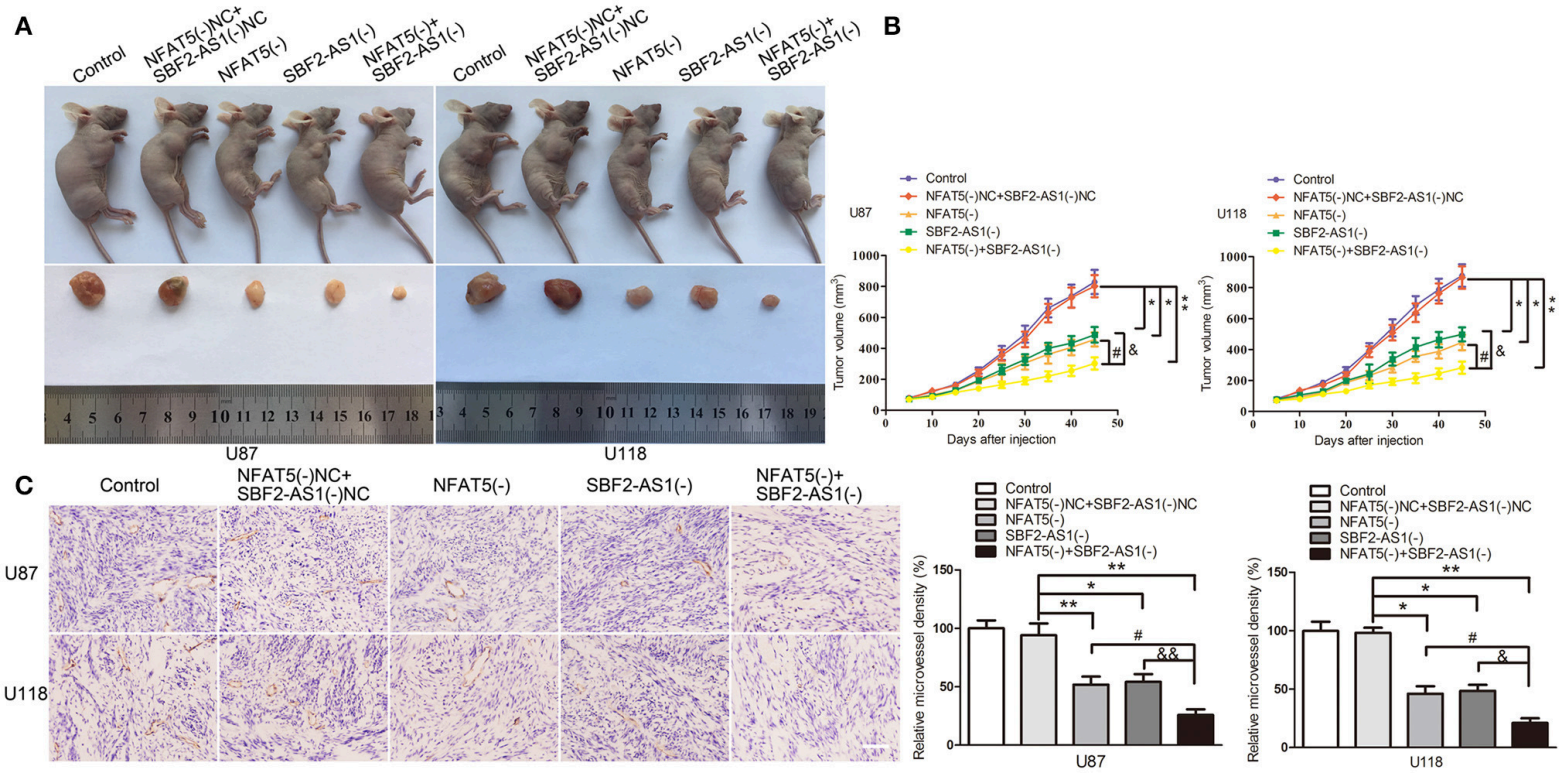
Combination of *NFAT5* knockdown and *SBF2-AS1* knockdown inhibited xenograft glioma growth and the microvessel density *in vivo*. The stable expressing U87 and U118 cells were used for the *in vivo* study. **(A)** The nude mice carrying tumors from respective groups and a sample tumor from each group were shown. **(B)** Xenograft glioma growth curves in nude mice. Tumor volume was measured every 5 days after injection. Data represent mean ± s.d. (*n* = 7, each group). **(C)** Tumors were harvested on day 45 and paraffin-embedded tumor sections were prepared. CD-31 immunohistochemistry staining was performed to determine the microvessel density (MVD). (magnification, × 400; scale bar = 50 μm). Data represent mean ± s.d. (*n* = 4, each group). ^*^P, ^&^*P*, and ^#^*P* < 0.05, ^&&^*P* and ^**^P < 0.01.

To define the roles of NFAT5 and SBF2-AS1 in tumor growth ability *in vivo*, we established mouse xenograft models with *NFAT5* knockdown, *SBF2-AS1* knockdown and *NFAT5* and *SBF2-AS1* dual-knockdown GBM cells by subcutaneous injection. Results showed that the volume of xenograft gliomas were dramatically reduced in NFAT5(-) group, SBF2-AS1(-) group, and NFAT5(-)+SBF2-AS1(-) group compared with NFAT5(-)NC+SBF2-AS1(-)NC group. The combination knockdown of *NFAT5* and *SBF2-AS1* produced the minimum xenograft glioma volume (Figures 10A,B).

CD31 immunohistochemistry staining was performed to determined the microvessel density of the xenograft gliomas. As shown in Figure 10C, downregulation of NFAT5 or SBF2-AS1 decreased the microvessel density of xenograft gliomas and *NFAT5* knockdown combined with *SBF2-AS1* knockdown presented the lowest microvessel density.

## Discussion

**Figure 11:**
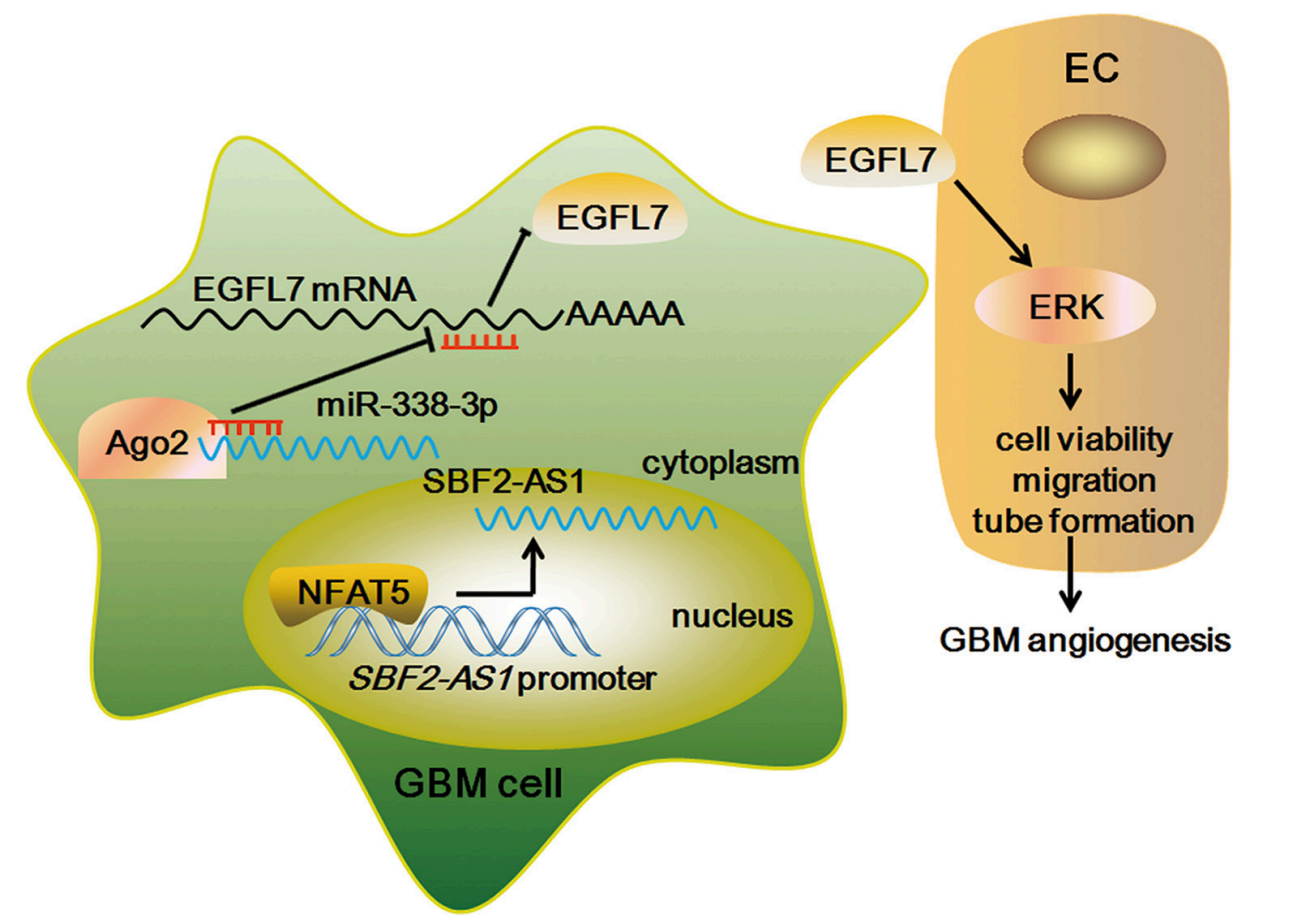
The Schematic representation of NFAT5/SBF2-AS1/miR-338-3p/EGFL7 axis in GBM cell-driven angiogenesis.

In this study, NFAT5 and SBF2-AS1 was highly expressed in glioma samples and positively associated with glioma WHO grades. Knockdown of *NFAT5* in GBM cell lines inhibited tumor-driven angiogenesis. Similarly, downregulation of SBF2-AS1 impaired GBM cell-driven angiogenesis. NFAT5 positively regulated SBF2-AS1 via binding to its promoter region in a sequence-dependent manner. In addition, SBF2-AS1 sponged miR-338-3p to release the post-translational inhibition of EGFL7, which stimulating the cell viability, migration and tube formation of endothelial cells (Figure 11). Furthermore, knockdown of both *NFAT5* and *SBF2-AS1* inhibited the xenograft glioma growth and reduced the microvessel density.

NFAT5 is a member of the nuclear factor of activated T cells (NFAT) transcription factors. Recent studies have pointed to an important role for NFAT5 in modulating tumor biology. Küper et al. (2014) found that knockdown of NFAT5 is accompanied by a significant decrease in the proliferation and migration of renal carcinoma cells. Guo et al. (Guo and Jin, 2015) reported that NFAT5 inhibition impairs the proliferation and migration of lung adenocarcinoma cells by downregulating aquaporin-5. In breast cancer, NFAT5 was demonstrated to be a putative biomarker of inflammatory breast cancer phenotype (Remo et al., 2015). Moreover, the expression of NFAT5 promotes breast cancer cells migration (Jauliac et al., 2002) and tumor-driven angiogenesis (Li et al., 2014). On the contrary, NFAT5 acts as a tumor suppressor in hepatocellular carcinoma (Qin et al., 2017). In this study, we found that NFAT5 was deregulated in GBM and knockdown of NFAT5 inhibited GBM cell-driven angiogenesis in *in vitro* and *in vivo* studies.

NFAT5 was shown to promote pro-angiogenic factor expression. Amara et al. (2016) clarified that NFAT5 interacts with STAT3, which stimulates VEGF-A expression via binding to its promoter region in breast cancer cells. Moreover, Veltmann et al. (2016) found that knockdown of *NFAT5* reduces bFGF transcripts in retinal pigment epithelium cells, which indicates that *bFGF* gene were transcriptionally activated by NFAT5 under hyperosmotic conditions. In addition, Madonna et al. (2016) demonstrated that high glucose induces human aortic endothelial cells angiogenesis and COX-2 expression possibly through the activation of NFAT5. In this study, downregulation of NFAT5 in GBM cell lines blocked the expression and secretion of EGFL7 via the SBF2-AS1/miR-338-3p axis.

An increasing number of studies have showed that aberrant expressed lncRNAs are vital regulatory factors in GBM. In particular, lncRNA H19 (Jiang et al., 2016), HULC (Zhu et al., 2016), and TUG1 (Cai et al., 2017) were demonstrated to regulate GBM angiogenesis. SBF2-AS1 is primarily associated with the poor prognosis in patients with non-small cell lung cancer (Zhao et al., 2016). In this study, we found that SBF2-AS1 was highly expressed in GBM and associated with GBM cell-driven angiogenesis. Mechanisms underlying the deregulated lncRNAs in tumors are under investigation. Zhang et al. (2017) uncovered that lncRNA Orilnc1 is regulated by RAS/RAF/MEK/ERK signaling via the transcriptional activation of AP1. Similar, Teng et al. (2016) reported that an increase in lncRNA HCP5 expression can be observed by the introduction of transcription factor RUNX1. In line with the above findings, our results clarified that NFAT5 blockade reduced the expression of SBF2-AS1, which possibly attributed to the absent of NFAT5 in *SBF2-AS1* promoter region. Recent publications indicated that RNA binding proteins regulate lncRNAs expression via stabilizing them (Chai et al., 2016; Wang L. et al., 2017). Whether RNA binding proteins are involved in NFAT5-regulated SBF2-AS1 expression change deserves further study.

The mechanisms of lncRNAs in tumor biology have not been elucidated very clearly. The well-researched function pattern for lncRNAs is acting as miRNA sponges to release the post-translational inhibition of target mRNAs. For instance, TUG1 maintains stemness features of glioma stem cells via sponging miR-145 to promote *SOX2* and *MYC* expression (Katsushima et al., 2016). ZFAS1 was reported to promote hepatocellular carcinoma metastasis via sponging miR-150 to activate ZEB1, MMP14, and MMP16 (Li et al., 2015). Consistent with the above findings, we demonstrated that SBF2-AS1 regulates EGFL7 may via sponging miR-338-3p.

miR-338-3p was verified to be a tumor suppressor in gastric cancer (Guo et al., 2014), hepatocellular carcinoma (Huang et al., 2011), and so forth. Moreover, miR-338-3p exerts anti-angiogenic effect in hepatocellular carcinoma (Zhang et al., 2016). In GBM, miR-338-3p was shown lowly expressed and restoration of miR-338-3p inhibited GBM cells malignant behaviors (Howe et al., 2017). In this study, our finding manifested that ectopic expression of miR-338-3p in GBM cells reduced GBM cell-driven angiogenesis. miRNAs regulate gene expression by degradation of target mRNAs or inhibition of translation (Bartel, 2004). In this study, we found that miR-338-3p reduced EGFL7 protein levels without affecting EGFL7 mRNA level, perhaps through the translation inhibition.

Previous studies have well-established the involvement of EGFL7 in endothelial cell behaviors. Knockdown of *EGFL7* in zebrafish embryos specifically blocks vascular tubulogenesis (Parker et al., 2004). EGFL7 also acts as a chemoattractant for embryonic endothelial cells migration (Campagnolo et al., 2005). In addition, EGFL7 protects endothelial cells from hyperoxia-induced cell death (Xu et al., 2008). Recent evidence focuses on the pro-angiogenic role of EGFL7 in cancer. Shen et al. (2016) reported that EGFL7 is high expressed in pancreatic carcinoma and promotes cancer invasion and angiogenesis. Hu et al. (2016) demonstrated that miR-126 inhibited hepatocellular carcinoma angiogenesis through reducing EGFL7. In addition, ERK activation plays an important role in EGFL7-mediated pro-angiogenic effect (Takeuchi et al., 2014; Chim et al., 2015; Gong et al., 2017). Increased EGFL7 levels promote glioma angiogenesis and may predict poor prognosis of GBM patients (Wang F. Y. et al., 2017). Consistent with the above findings, we demonstrated that enhanced EGFL7 levels in GBM cells promoted ECs cell viability, migration, and tube formation via activating ERK.

GBM angiogenesis is a complicated process. Endothelial cell-dependent manner, which was focused on in this study, plays a predominant role (Zhang et al., 2015; Gilbert, 2016). Moreover, macrophages in the tumor microenvironment are key regulators of GBM angiognensis (Lu-Emerson et al., 2013; Zhu et al., 2017). Recent evidence indicated that vasculogenic mimicry, which is characterized as endothelium-independent tubular structure, may be a novel blood supply manner in malignant glioma (Yue and Chen, 2005; Liu et al., 2011). In addition, glioblastoma stem-like cells could differentiate into endothelial cells, which promotes the angiogenetic malignancy (Chroscinski et al., 2015). An interesting line of investigation would be to evaluate whether NFAT5 is involved in the above cellular process.

In conclusion, increased NFAT5 levels were positively correlated with glioma pathological grade. Knockdown of *NFAT5* inhibited GBM cell-driven angiogenesis by reducing the expression and secretion of EGFL7 via SBF2-AS1/miR-338-3p axis. NFAT5/SBF2-AS1/miR-338-3p/EGFL7 pathway may lead to better anti-angiogenic strategies for the treatment of GBM.

## Author contributions

YL and YX conceived and designed this study. HY, XL and JZ performed the main experiments. LZ and ZL helped with the Immunohistochemistry assay. SS and LZ helped with the animal experiments. JZ, SS, and ZL collected the clinical data and analyzed the data. HY and XL drafted the manuscript and performed the literature review. YL and YX revised the article critically. All authors had final approval of the submitted versions.

### Conflict of interest statement

The authors declare that the research was conducted in the absence of any commercial or financial relationships that could be construed as a potential conflict of interest.

## References

[B1] Amara S. Alotaibi D. Tiriveedhi V. (2016). NFAT5/STAT3 interaction mediates synergism of high salt with IL-17 towards induction of VEGF-A expression in breast cancer cells. Oncol. Lett. 12, 933–943. 10.3892/ol.2016.4713 27446373 PMC4950837

[B2] Bartel D. P. (2004). MicroRNAs: genomics, biogenesis, mechanism, and function. Cell 116, 281–297. 10.1016/S0092-8674(04)00045-5 14744438

[B3] Beckedorff F. C. Amaral M. S. Deocesano-Pereira C. Verjovski-Almeida S. (2013). Long non-coding RNAs and their implications in cancer epigenetics. Biosci. Rep. 33:e00061. 10.1042/BSR20130054 23875687 PMC3759304

[B4] Cai H. Liu X. Zheng J. Xue Y. Ma J. Li Z.. (2017). Long non-coding RNA taurine upregulated 1 enhances tumor-induced angiogenesis through inhibiting microRNA-299 in human glioblastoma. Oncogene 36, 318–331. 10.1038/onc.2016.212 27345398

[B5] Campagnolo L. Leahy A. Chitnis S. Koschnick S. Fitch M. J. Fallon J. T.. (2005). EGFL7 is a chemoattractant for endothelial cells and is up-regulated in angiogenesis and arterial injury. Am. J. Pathol. 167, 275–284. 10.1016/S0002-9440(10)62972-0 15972971 PMC1451775

[B6] Chai Y. Liu J. Zhang Z. Liu L. (2016). HuR-regulated lncRNA NEAT1 stability in tumorigenesis and progression of ovarian cancer. Cancer Med. 5, 1588–1598. 10.1002/cam4.710 27075229 PMC4944886

[B7] Chim S. M. Kuek V. Chow S. T. Lim B. S. Tickner J. Zhao J.. (2015). EGFL7 is expressed in bone microenvironment and promotes angiogenesis via ERK, STAT3, and integrin signaling cascades. J. Cell. Physiol. 230, 82–94. 10.1002/jcp.24684 24909139

[B8] Chroscinski D. Sampey D. Maherali N. Reproducibility Project: Cancer Biology Reproducibility Project: Cancer Biology. (2015). Registered report: tumour vascularization via endothelial differentiation of glioblastoma stem-like cells. Elife 4:e04363. 10.7554/eLife.04363 25714925 PMC4383241

[B9] Gilbert M. R. (2016). Antiangiogenic therapy for glioblastoma: complex biology and complicated results. J. Clin. Oncol. 34, 1567–1569. 10.1200/JCO.2016.66.5364 27001588

[B10] Gong C. Fang J. Li G. Liu H. H. Liu Z. S. (2017). Effects of microRNA-126 on cell proliferation, apoptosis and tumor angiogenesis via the down-regulating ERK signaling pathway by targeting EGFL7 in hepatocellular carcinoma. Oncotarget 8, 52527–52542. 10.18632/oncotarget.17283 28881749 PMC5581048

[B11] Guo B. Liu L. Yao J. Ma R. Chang D. Li Z.. (2014). miR-338-3p suppresses gastric cancer progression through a PTEN-AKT axis by targeting P-REX2a. Mol. Cancer Res. 12, 313–321. 10.1158/1541-7786.MCR-13-0507 24375644

[B12] Guo K. Jin F. (2015). NFAT5 promotes proliferation and migration of lung adenocarcinoma cells in part through regulating AQP5 expression. Biochem. Biophys. Res. Commun. 465, 644–649. 10.1016/j.bbrc.2015.08.078 26299924

[B13] Howe J. R. T. Li E. S. Streeter S. E. Rahme G. J. Chipumuro E. Russo G. B.. (2017). MiR-338-3p regulates neuronal maturation and suppresses glioblastoma proliferation. PLoS ONE 12:e0177661 10.1371/journal.pone.0177661 28493990 PMC5426787

[B14] Hu M. H. Ma C. Y. Wang X. M. Ye C. D. Zhang G. X. Chen L.. (2016). MicroRNA-126 inhibits tumor proliferation and angiogenesis of hepatocellular carcinoma by down-regulating EGFL7 expression. Oncotarget 7, 66922–66934. 10.18632/oncotarget.11877 27611944 PMC5341847

[B15] Huang X. H. Chen J. S. Wang Q. Chen X. L. Wen L. Chen L. Z.. (2011). miR-338-3p suppresses invasion of liver cancer cell by targeting smoothened. J. Pathol. 225, 463–472. 10.1002/path.2877 21671467

[B16] Jain R. K. di Tomaso E. Duda D. G. Loeffler J. S. Sorensen A. G. Batchelor T. T. (2007). Angiogenesis in brain tumours. Nat. Rev. Neurosci. 8, 610–622. 10.1038/nrn2175 17643088

[B17] Jauliac S. Lopez-Rodriguez C. Shaw L. M. Brown L. F. Rao A. Toker A. (2002). The role of NFAT transcription factors in integrin-mediated carcinoma invasion. Nat. Cell Biol. 4, 540–544. 10.1038/ncb816 12080349

[B18] Jiang X. Yan Y. Hu M. Chen X. Wang Y. Dai Y.. (2016). Increased level of H19 long noncoding RNA promotes invasion, angiogenesis, and stemness of glioblastoma cells. J. Neurosurg. 2016, 129–136. 10.3171/2014.12.JNS1426.test 28306408

[B19] Katsushima K. Natsume A. Ohka F. Shinjo K. Hatanaka A. Ichimura N.. (2016). Targeting the Notch-regulated non-coding RNA TUG1 for glioma treatment. Nat. Commun. 7:13616. 10.1038/ncomms13616 27922002 PMC5150648

[B20] Kultz D. Csonka L. (1999). What sets the TonE during osmotic stress? Proc. Natl. Acad. Sci. U.S.A. 96, 1814–1816. 10.1073/pnas.96.5.1814 10051549 PMC33524

[B21] Küper C. Beck F. X. Neuhofer W. (2014). NFAT5-mediated expression of S100A4 contributes to proliferation and migration of renal carcinoma cells. Front. Physiol. 5:293. 10.3389/fphys.2014.00293 25152734 PMC4126233

[B22] Leon S. P. Folkerth R. D. Black P. M. (1996). Microvessel density is a prognostic indicator for patients with astroglial brain tumors. Cancer 77, 362–372. 10.1002/(SICI)1097-0142(19960115)77:2<362::AID-CNCR20>3.0.CO;2-Z 8625246

[B23] Li J. T. Wang L. F. Zhao Y. L. Yang T. Li W. Zhao J.. (2014). Nuclear factor of activated T cells 5 maintained by hotair suppression of miR-568 upregulates S100 calcium binding protein A4 to promote breast cancer metastasis. Breast Cancer Res. 16:454. 10.1186/s13058-014-0454-2 25311085 PMC4303133

[B24] Li T. Xie J. Shen C. Cheng D. Shi Y. Wu Z.. (2015). Amplification of long noncoding RNA ZFAS1 promotes metastasis in hepatocellular carcinoma. Cancer Res. 75, 3181–3191. 10.1158/0008-5472.CAN-14-3721 26069248

[B25] Liu X. M. Zhang Q. P. Mu Y. G. Zhang X. H. Sai K. Pang J. C.. (2011). Clinical significance of vasculogenic mimicry in human gliomas. J. Neurooncol. 105, 173–179. 10.1007/s11060-011-0578-5 21533525 PMC3198193

[B26] Lopez-Rodriguez C. Antos C. L. Shelton J. M. Richardson J. A. Lin F. Novobrantseva T. I.. (2004). Loss of NFAT5 results in renal atrophy and lack of tonicity-responsive gene expression. Proc. Natl. Acad. Sci. U.S.A. 101, 2392–2397. 10.1073/pnas.0308703100 14983020 PMC356961

[B27] Lu-Emerson C. Snuderl M. Kirkpatrick N. D. Goveia J. Davidson C. Huang Y.. (2013). Increase in tumor-associated macrophages after antiangiogenic therapy is associated with poor survival among patients with recurrent glioblastoma. Neurooncology 15, 1079–1087. 10.1093/neuonc/not082 23828240 PMC3714160

[B28] Lv J. Qiu M. Xia W. Liu C. Xu Y. Wang J.. (2016). High expression of long non-coding RNA SBF2-AS1 promotes proliferation in non-small cell lung cancer. J. Exp. Clin. Cancer Res. 35:75. 10.1186/s13046-016-0352-9 27154193 PMC4859961

[B29] Madonna R. Giovannelli G. Confalone P. Renna F. V. Geng Y. J. De Caterina R. (2016). High glucose-induced hyperosmolarity contributes to COX-2 expression and angiogenesis: implications for diabetic retinopathy. Cardiovasc. Diabetol. 15:18. 10.1186/s12933-016-0342-4 26822858 PMC4731895

[B30] Parker L. H. Schmidt M. Jin S. W. Gray A. M. Beis D. Pham T.. (2004). The endothelial-cell-derived secreted factor Egfl7 regulates vascular tube formation. Nature 428, 754–758. 10.1038/nature02416 15085134

[B31] Qin X. Wang Y. Li J. Xiao Y. Liu Z. (2017). NFAT5 inhibits invasion and promotes apoptosis in hepatocellular carcinoma associated with osmolality. Neoplasma 64, 502–510. 10.4149/neo_2017_403 28485155

[B32] Remo A. Simeone I. Pancione M. Parcesepe P. Finetti P. Cerulo L.. (2015). Systems biology analysis reveals NFAT5 as a novel biomarker and master regulator of inflammatory breast cancer. J. Transl. Med. 13:138. 10.1186/s12967-015-0492-2 25928084 PMC4438533

[B33] Shen X. Han Y. Xue X. Li W. Guo X. Li P.. (2016). Epidermal growth factor-like domain 7 promotes cell invasion and angiogenesis in pancreatic carcinoma. Biomed. Pharmacother. 77, 167–175. 10.1016/j.biopha.2015.12.009 26796281

[B34] Takeuchi K. Yanai R. Kumase F. Morizane Y. Suzuki J. Kayama M.. (2014). EGF-like-domain-7 is required for VEGF-induced Akt/ERK activation and vascular tube formation in an *ex vivo* angiogenesis assay. PLoS ONE 9:e91849. 10.1371/journal.pone.0091849 24647208 PMC3960138

[B35] Tay Y. Rinn J. Pandolfi P. P. (2014). The multilayered complexity of ceRNA crosstalk and competition. Nature 505, 344–352. 10.1038/nature12986 24429633 PMC4113481

[B36] Teng H. Wang P. Xue Y. Liu X. Ma J. Cai H.. (2016). Role of HCP5-miR-139-RUNX1 feedback loop in regulating malignant behavior of glioma cells. Mol. Ther. 24, 1806–1822. 10.1038/mt.2016.103 27434586 PMC5112034

[B37] Veltmann M. Hollborn M. Reichenbach A. Wiedemann P. Kohen L. Bringmann A. (2016). Osmotic induction of angiogenic growth factor expression in human retinal pigment epithelial cells. PLoS ONE 11:e0147312. 10.1371/journal.pone.0147312 26800359 PMC4723123

[B38] Wang F. Y. Kang C. S. Wang-Gou S. Y. Huang C. H. Feng C. Y. Li X. J. (2017). EGFL7 is an intercellular EGFR signal messenger that plays an oncogenic role in glioma. Cancer Lett. 384, 9–18. 10.1016/j.canlet.2016.10.009 27725228

[B39] Wang L. Ye S. Wang J. Gu Z. Zhang Y. Zhang C.. (2017). HuR stabilizes lnc-Sox5 mRNA to promote tongue carcinogenesis. Biochemistry 82, 438–445. 10.1134/S0006297917040046 28371600

[B40] Weidner N. Semple J. P. Welch W. R. Folkman J. (1991). Tumor angiogenesis and metastasis–correlation in invasive breast carcinoma. N. Engl. J. Med. 324, 1–8. 10.1056/NEJM199101033240101 1701519

[B41] Wen P. Y. Kesari S. (2008). Malignant gliomas in adults. N. Engl. J. Med. 359, 492–507. 10.1056/NEJMra0708126 18669428

[B42] Woo S. K. Lee S. D. Na K. Y. Park W. K. Kwon H. M. (2002). TonEBP/NFAT5 stimulates transcription of HSP70 in response to hypertonicity. Mol. Cell. Biol. 22, 5753–5760. 10.1128/MCB.22.16.5753-5760.2002 12138186 PMC133967

[B43] Wurdinger T. Tannous B. A. Saydam O. Skog J. Grau S. Soutschek J.. (2008). miR-296 regulates growth factor receptor overexpression in angiogenic endothelial cells. Cancer Cell 14, 382–393. 10.1016/j.ccr.2008.10.005 18977327 PMC2597164

[B44] Xu D. Perez R. E. Ekekezie I. I. Navarro A. Truog W. E. (2008). Epidermal growth factor-like domain 7 protects endothelial cells from hyperoxia-induced cell death. Am. J. Physiol. Lung Cell. Mol. Physiol. 294, L17–L23. 10.1152/ajplung.00178.2007 17934064

[B45] Yu H. Xue Y. Wang P. Liu X. Ma J. Zheng J.. (2017). Knockdown of long non-coding RNA XIST increases blood-tumor barrier permeability and inhibits glioma angiogenesis by targeting miR-137. Oncogenesis 6:e303. 10.1038/oncsis.2017.7 28287613 PMC5533948

[B46] Yue W. Y. Chen Z. P. (2005). Does vasculogenic mimicry exist in astrocytoma? J. Histochem. Cytochem. 53, 997–1002. 10.1369/jhc.4A6521.2005 15923371

[B47] Zhang D. Zhang G. Hu X. Wu L. Feng Y. He S. (2017). Oncogenic RAS regulates long non-coding RNA Orilnc1 in human cancer. Cancer Res. 77, 3745–3757. 10.1158/0008-5472.CAN-16-1768 28473531 PMC5511552

[B48] Zhang M. Ye G. Li J. Wang Y. (2015). Recent advance in molecular angiogenesis in glioblastoma: the challenge and hope for anti-angiogenic therapy. Brain Tumor Pathol. 32, 229–236. 10.1007/s10014-015-0233-5 26437643

[B49] Zhang T. Liu W. Zeng X. C. Jiang N. Fu B. S. Guo Y.. (2016). Down-regulation of microRNA-338-3p promoted angiogenesis in hepatocellular carcinoma. Biomed. Pharmacother. 84, 583–591. 10.1016/j.biopha.2016.09.056 27694002

[B50] Zhang Y. Wang X. Xu B. Wang B. Wang Z. Liang Y.. (2013). Epigenetic silencing of miR-126 contributes to tumor invasion and angiogenesis in colorectal cancer. Oncol. Rep. 30, 1976–1984. 10.3892/or.2013.2633 23900443

[B51] Zhao Q. S. Li L. Zhang L. Meng X. W. Li L. L. Ge X. F.. (2016). Over-expression of lncRNA SBF2-AS1 is associated with advanced tumor progression and poor prognosis in patients with non-small cell lung cancer. Eur. Rev. Med. Pharmacol. Sci. 20, 3031–3034. 27460731

[B52] Zhu C. Chrifi I. Mustafa D. M. van der Weiden M. Leenen P. J. Duncker D. J.. (2017). CECR1-mediated cross talk between macrophages and vascular mural cells promotes neovascularization in malignant glioma. Oncogene. [Epub ahead of print]. 10.1038/onc.2017.145 28534507 PMC5611481

[B53] Zhu Y. Zhang X. Qi L. Cai Y. Yang P. Xuan G.. (2016). HULC long noncoding RNA silencing suppresses angiogenesis by regulating ESM-1 via the PI3K/Akt/mTOR signaling pathway in human gliomas. Oncotarget 7, 14429–14440. 10.18632/oncotarget.7418 26894862 PMC4924726

