# Supplementary material for: Transcription Factor NFAT5 Promotes Glioblastoma Cell-driven Angiogenesis via SBF2-AS1/miR-338-3p-Mediated EGFL7 Expression Change
Source: Front Mol Neurosci. 2017 Sep 21;10:301. doi: 10.3389/fnmol.2017.00301 (PMC5613209; doi:10.3389/fnmol.2017.00301)
Supplement: Supplementary file 1 [file Table1.PDF]

Table S1. Primers and probes used for RT-qPCR.

Primer or Probe	Gene	Sequence (5'->3') or Assay ID
Primer	NFAT5	F: GTCACCGACAGCAAGGCTAT
		R: AAGACTGTGTGCCTCTTCGG
	SBF2-AS1	F: CCACGACCCAGAAGGAGTCT
		R: GCATTGATGGAGCATTGCGA
	EGFL7	F: TGTGGAGCAGCAATATGCCA
		R: CCCCTCCTAGCACTGCATTC
	GAPDH	F: CCCATCACCATCTTCCAGGAG
		R: GTTGTCATGGATGACCTTGGC
Probe	miR-338-3p	002252(Applied biosystems)
	U6	001973(Applied biosystems)
