# Supplementary material for: Transcription Factor NFAT5 Promotes Glioblastoma Cell-driven Angiogenesis via SBF2-AS1/miR-338-3p-Mediated EGFL7 Expression Change
Source: Front Mol Neurosci. 2017 Sep 21;10:301. doi: 10.3389/fnmol.2017.00301 (PMC5613209; doi:10.3389/fnmol.2017.00301)
Supplement: Supplementary file 2 [file Table2.PDF]

Table S2. shRNA target sequences

Gene	Sequence(5'->3')
NFAT5	CACTGAGGTACCTCGTAAATC
SBF2-AS1	GCTGAGTTAATCAGAGTTATG
NC	GTTCTCCGAACGTGTCACGT
