# Supplementary material for: Transcription Factor NFAT5 Promotes Glioblastoma Cell-driven Angiogenesis via SBF2-AS1/miR-338-3p-Mediated EGFL7 Expression Change
Source: Front Mol Neurosci. 2017 Sep 21;10:301. doi: 10.3389/fnmol.2017.00301 (PMC5613209; doi:10.3389/fnmol.2017.00301)
Supplement: Supplementary file 3 [file Table3.PDF]

Table S3. Primers used for ChIP experiments

Gene	Binding site or Control	Sequence (5'->3')	Product size (bp)	Annealing temperature (℃)
EGFL7	PCR1	F: AAGGTCTGTGCCATGATCCC	149	54
		R: GGTATGGGTGGAGCACACTC		
	PCR2	F: TAGAGGGGAGCTGGTTCCTG	190	59
		R: GGACACCCCCATTTCCCTG		
SBF2-AS1	PCR1	F: TGCTGTTCATTACACCACCCA	114	56
		R: TATGTAGGGGTGGCACATGG		
	PCR2	F: CCTGAGGCACCTGGTGTTTT	167	55
		R: TAGCCTTGAATGGCTGACCA		
	PCR3	F: ACTTAATGGGCCTGTCGCTG	166	57
		R: CCTTTGCAGGACACCTCAGA		
	PCR4	F: TGATAGGCCAATTCGTCCCC	245	56
		R: CTGGAAGGCTGTGCACTGAA		
