# Supplementary material for: Transcription Factor NFAT5 Promotes Glioblastoma Cell-driven Angiogenesis via SBF2-AS1/miR-338-3p-Mediated EGFL7 Expression Change
Source: Front Mol Neurosci. 2017 Sep 21;10:301. doi: 10.3389/fnmol.2017.00301 (PMC5613209; doi:10.3389/fnmol.2017.00301)
Supplement: Supplementary file 4 [file Image1.PDF]

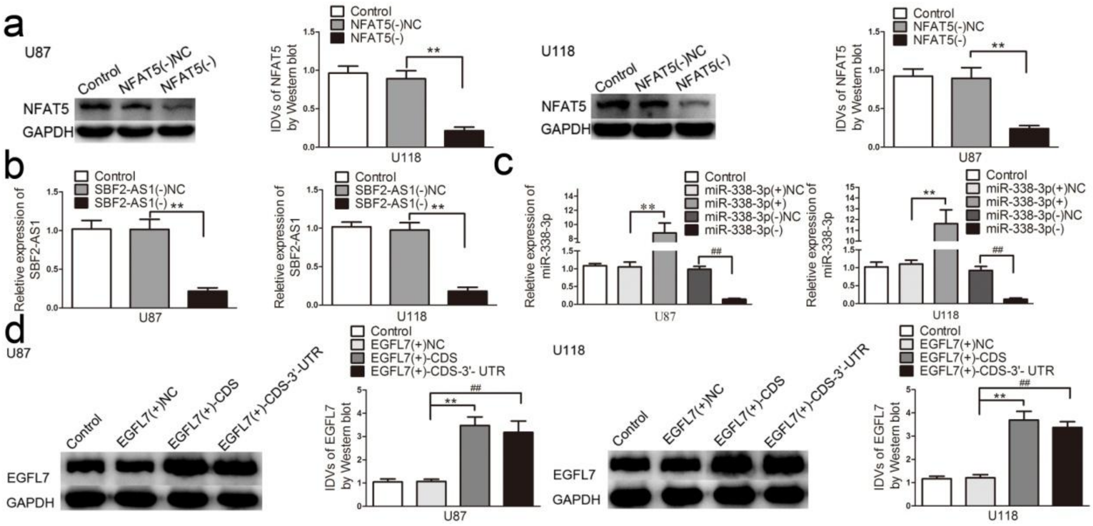

Figure S1. Tansfection efficiency of NFAT5, SBF2-AS1, miR-338-3p and EGFL7. (a) Relative NFAT5 levels in U87 and U118 cells transfected shNFAT5. Data represent mean ± s.d. (n=3, each). $^{**}P$<0.01 (b) Relative SBF2-AS1 levels in U87 and U118 cells transfected shSBF2-AS1. Data represent mean ± s.d. (n=4, each), $^{**}P$<0.01. (c) Relative miR-338-3p levels in U87 and U118 cells transfected agomir-338-3p or antagomir-338-3p. Data represent mean ± s.d. (n=4, each), $^{**}P$ and $^{\#\#}P$<0.01. (d) Relative EGFL7 levels in U87 and U118 cells transfected pIRES2-EGFL7-CDS or pIRES2-EGFL7-CDS-3'-UTR. Data represent mean ± s.d. (n=3, each). $^{**}P$ and $^{\#\#}P$<0.01.

Table S1. Primers and probes used for RT-qPCR.

Primer or Probe	Gene	Sequence (5'->3') or Assay ID
Primer	NFAT5	F: GTCACCGACAGCAAGGCTAT
		R: AAGACTGTGTGCCTCTTCGG
	SBF2-AS1	F: CCACGACCCAGAAGGAGTCT
		R: GCATTGATGGAGCATTGCGA
	EGFL7	F: TGTGGAGCAGCAATATGCCA
		R: CCCCTCCTAGCACTGCATTC
	GAPDH	F: CCCATCACCATCTTCCAGGAG
		R: GTTGTCATGGATGACCTTGGC
Probe	miR-338-3p	002252(Applied biosystems)
	U6	001973(Applied biosystems)

Table S2. shRNA target sequences

Gene	Sequence(5'->3')
NFAT5	CACTGAGGTACCTCGTAAATC
SBF2-AS1	GCTGAGTTAATCAGAGTTATG
NC	GTTCTCCGAACGTGTCACGT

Table S3. Primers used for ChIP experiments

Gene	Binding site or Control	Sequence (5'->3')	Product size (bp)	Annealing temperature (℃)
EGFL7	PCR1	F: AAGGTCTGTGCCATGATCCC	149	54
		R: GGTATGGGTGGAGCACACTC		
	PCR2	F: TAGAGGGGAGCTGGTTCCTG	190	59
		R: GGACACCCCCATTTCCCTG		
SBF2-AS1	PCR1	F: TGCTGTTCATTACACCACCCA	114	56
		R: TATGTAGGGGTGGCACATGG		
	PCR2	F: CCTGAGGCACCTGGTGTTTT	167	55
		R: TAGCCTTGAATGGCTGACCA		
	PCR3	F: ACTTAATGGGCCTGTCGCTG	166	57
		R: CCTTTGCAGGACACCTCAGA		
	PCR4	F: TGATAGGCCAATTCGTCCCC	245	56
		R: CTGGAAGGCTGTGCACTGAA		
