# Supplementary material for: Transcription Factor NFAT5 Promotes Glioblastoma Cell-driven Angiogenesis via SBF2-AS1/miR-338-3p-Mediated EGFL7 Expression Change
Source: Front Mol Neurosci. 2017 Sep 21;10:301. doi: 10.3389/fnmol.2017.00301 (PMC5613209; doi:10.3389/fnmol.2017.00301)
Supplement: Supplementary file 5 [file Image2.PDF]

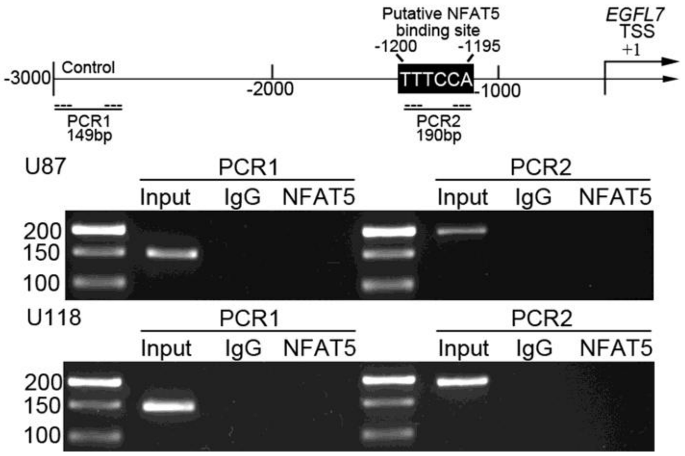

Figure S2. Schematic representation of the human *EGFL7* promoter region. Chromatin immunoprecipitation PCR products for putative NFAT5-binding sites and an upstream region not expected to associate with NFAT5 are amplified by PCR using their specific primers (n=3).
