# Supplementary material for: Transcription Factor NFAT5 Promotes Glioblastoma Cell-driven Angiogenesis via SBF2-AS1/miR-338-3p-Mediated EGFL7 Expression Change
Source: Front Mol Neurosci. 2017 Sep 21;10:301. doi: 10.3389/fnmol.2017.00301 (PMC5613209; doi:10.3389/fnmol.2017.00301)
Supplement: Supplementary file 6 [file Image3.PDF]

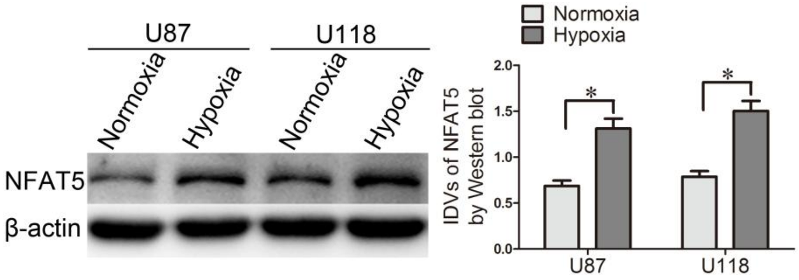

Figure S3. NFAT5 was upregulated under hypoxia conditions in U87 and U118 cells. Relative NFAT5 levels under the normoxia or hypoxia environment were determined by Western blot in U87 and U118 cells. Data represent mean ± s.d. (n=3, each). $^{*}P<0.05$.
